# Otoliths of Caspian gobies (Teleostei: Gobiidae): Morphological diversity and phylogenetic implications

**DOI:** 10.1371/journal.pone.0285857

**Published:** 2023-05-15

**Authors:** Fatah Zarei, Hamid Reza Esmaeili, Carol A. Stepien, Marcelo Kovačić, Keyvan Abbasi

**Affiliations:** 1 Ichthyology and Molecular Systematics Research Laboratory, Zoology Section, Department of Biology, School of Science, Shiraz University, Shiraz, Iran; 2 National Museum of Natural History, Department of Vertebrate Zoology, Smithsonian Institution, Washington D.C., United States of America; 3 Natural History Museum Rijeka, Rijeka, Croatia; 4 Inland Waters Aquaculture Research Center, Iranian Fisheries Sciences Research Institute, Agricultural Research, Education and Extension Organization, Bandar Anzali, Iran; Laboratoire de Biologie du Développement de Villefranche-sur-Mer, FRANCE

## Abstract

Otoliths (ear stones) of the inner ears of teleost fishes, which develop independently from the skeleton and are functionally associated with hearing and the sense of equilibrium, have significantly contributed to contemporary understanding of teleost fish systematics and evolutionary diversity. The sagittal otolith is of particular interest, since it often possesses distinctive morphological features that differ significantly among species, and have been shown to be species- and genus-specific, making it an informative taxonomic tool for ichthyologists. The otolith morphology of the Caspian Sea gobiids has not been thoroughly studied yet, with data available for only a few species. The aim of the present paper is to examine the qualitative and quantitative taxonomic and phylogenetic information in the sagittal otoliths of these species. A total of 118 otoliths representing 30 gobiid species (including 53.5% of the Caspian gobiofauna) in three gobiid lineages (i.e., *Gobius*, *Pomatoschistus*, and *Acanthogobius*) and 11 genera (i.e., all Ponto-Caspian gobiid genera except *Babka*) were analysed at taxonomic levels using an integrated descriptive and morphometric approach. The results indicated high taxonomic efficiency of otolith morphology and morphometry at taxonomic levels for the Ponto-Caspian gobiids. Our qualitative and quantitative otolith data also (i) support the monophyly of neogobiin gobies, (ii) along with other morphological and ecological data, offer a new perspective on the systematics of *Neogobius bathybius*, (iii) suggest the reassignment of *Hyrcanogobius bergi* to the genus *Knipowitschia*, and (iv) question the phylogenetic integrity of the four phenotypic groups previously defined in the tadpole-goby genus *Benthophilus*; however, more studies are needed to complete these evaluations and confirm our otolith study findings.

## Introduction

The otoliths of the inner ears in teleost fishes are arranged in three pairs termed the saccular (sagittae, the largest otoliths in most teleosts), utricular (lapilli), and lagenar (asterisci) otoliths. They are aragonitic mineralizations that develop independently from the skeleton, which are functionally associated with the senses of hearing and equilibrium [1]. Analysis of the shapes of otoliths has significantly contributed to knowledge of teleost systematics and biodiversity [2–7], as well as historic diversity, phylogeny, zoogeography, and climatology [8–12], ancient and modern fisheries [13], life history and habitat [14], and population structure [6, 7, 15]. The sagittal otolith (referred to as otolith hereforth) is of particular interest, since it often possesses distinctive morphological features that vary among populations, species, and genera [10, 16–18]. Some studies have concluded that otolith shape is primarily defined by genetic factors [19–21], however, others suggest that while genetics constrain the overall otolith’s shape, environmental conditions often alter its somatic growth rates, which can affect the otolith’s shape and result in intraspecific variation across the species’ distribution [22–26]. The extent to which otolith shape variations are genetically or environmentally induced are controversial and may differ among taxa.

The Ponto-Caspian basin, comprising the Black Sea, Sea of Azov, and Caspian Sea basins, has been the evolutionary stage for two main lineages of Gobiidae *sensu* Gill & Mooi [27]: (i) the endemic Ponto-Caspian benthophiline gobiids belonging to the *Gobius* lineage [28], classified in two main groups, nine genera, and three tribes: (1) the neogobiin group divided in the tribes Neogobiini (containing the genus *Neogobius* Iljin, 1927) and Ponticolini (four genera: *Ponticola* Iljin, 1927, *Mesogobius* Bleeker, 1874, *Proterorhinus* Smitt, 1899, and *Babka* Iljin, 1927), and (2) the benthophilin or tadpole gobies group comprising the tribe Benthophilini (four genera: *Anatirostrum* Iljin, 1930, *Benthophilus* Eichwald, 1831, *Benthophiloides* Beling & Iljin, 1927, and *Caspiosoma* Iljin, 1927. These tribes follow recommendations by Neilson and Stepien [29] and Agorreta et al. [28]. (ii) A branch of the *Pomatoschistus* lineage [28] or sand gobies, represented in the Ponto-Caspian basin by several species of *Knipowitschia* Iljin, 1927 and the monotypic genus *Hyrcanogobius* Iljin, 1928 [30].

Gobiid fauna of the Caspian Sea basin now include 43 species in 12 genera. Of these, 35 species are endemic to the Caspian Sea basin, seven are native to the Ponto-Caspian overall, and one species is exotic. The North, Middle and South Caspian Sea sub-basins harbor 21, 31, and 38 species, respectively, of which zero, three, and 10 are endemic to each [31]. Due to their low economic importance, small sizes, and distributions in difficult-to-sample deeper-water habitats [32–34], little is known about their biological characteristics and ecology. However, Gobiidae comprise 1/4 of species native to the Caspian Sea [31] and insights into their diversification and adaptations may help elucidate ecological and biogeographic understanding about the patterns and processes of species flock formation in the Ponto-Caspian realm.

Since the mid-2000s, Caspian Sea gobiid studies have examined the molecular phylogeny and biogeography of a limited number of species [7, 29, 30, 35], with several new species described in recent years [5, 36–39]. On the other hand, detailed discussions of their morphological characters have become rare, and these molecular-based studies suggest phylogenetic relationships that have not been considered with morphological information and therewith inspire new comparative studies to test those relationships. Hitherto, otolith morphology has been studied for just a few species [5–7, 38, 40, 45]. This study, using a taxonomically broad sample, aims to compare the otolith morphology of extant Caspian gobiids at different taxonomic levels based on detailed descriptions and morphometric analysis to reveal the taxonomic and phylogenetic information inscribed in their otoliths. The results not only provide another step towards the total-evidence phylogeny of the extant Pono-Caspian gobiids, but will also be beneficial for all future studies dealing with the taxonomic and phylogenetic assignment of fossil otolith records and otolith-based gobiid taxa described from the Ponto-Caspian basin and thus will help to improve our understanding of the pattern and process of gobiid diversification in the region.

## Materials and methods

### Sampling, otolith dissection, and SEM imaging

Necessary permits for sampling and observational field studies in the Southern Caspian Sea sub-basin have been obtained by the authors from the Iranian Department of Environment and the Iranian Fisheries Science Research Institute. New gobiid specimens were collected from the Southern Caspian Sea sub-basin using different methods, euthanized with an overdose of quinaldine sulfate and fixed in 70% ethanol, labeled, and deposited in the Zoological Museum of Shiraz University (ZM-CBSU). Fish sampling was approved by the Ethic Committee of Biology Department, Shiraz University (SU-9630190). Taxonomic identifications were accomplished using available keys, primary taxonomic literature, and DNA sequence analysis [5, 7, 30, 31, 33–35, 39, 41]. The skull of each specimen was opened dorsally with a scalpel and the left sagittal otolith was extracted using fine forceps, cleaned in 5% KOH solution (~5 min), washed in distilled water (~5 min), and dried at room temperature. The otoliths were coated with gold [42], and SEM images were taken using a VEGA3 TESCAN at Shiraz University.

The original Caspian Sea material comprised otoliths isolated from 92 specimens belonging to 17 Ponto-Caspian endemics and one introduced species from the genus *Rhinogobius* Gill, 1859 (Table 1). The original Black Sea basin material comprised eight otoliths isolated from two species, *Proterorhinus semilunaris* (Heckel, 1837) and *Mesogobius batrachocephalus* (Pallas, 1814) (Table 1). In addition to the original material, otolith photographs of 14 extant species from the Ponto-Caspian basin were recycled from previous publications [12, 38, 43–45; Table 1]. Thus, a total of 118 otoliths representing three lineages, 11 genera (i.e., all Ponto-Caspian endemic gobiid genera except *Babka*), and 30 species were compiled. The 30 species included 17 species that are Caspian Sea endemics, six species that are Ponto-Caspian natives, two species that are Azov Sea endemics, three Black Sea/Azov Sea endemics, and one exotic species. Accordingly, this study includes otoliths from 23 species having distributions in the Caspian Sea basin, totaling 53.5% of the known Caspian Sea gobiofauna [31].

**Table 1:** The original and published otolith material used in this study. Status: presence status in the Ponto-Caspian basin, i.e., CE, Caspian Sea endemic; PCN, Ponto-Caspian native; AZE, Azov Sea endemic; BAN, Black Sea/Azov Sea native; BE, Black Sea endemic; EX, exotic. Quant.: taxonomic level of quantitative analysis, i.e., L, lineage; T, tribe; G, genus; S, species. N, number of specimens; SL, standard length in mm. Institutional abbreviations: CMNFI, Canadian Museum of Nature in Ottawa; NMNH NASU, Zoological Museum of Ukraine in Kiev; RBINS, Royal Belgian Institute of Natural Sciences; SMF, Senckenberg Museum, Frankfurt/Main; ZM-CBSU, Zoological Museum of Shiraz University, Collection of Biology Department, Shiraz, Iran; ZMMU/ZMMSU, Zoological Mueum, Moscow University in Moscow; ZMUC, Zoological Museum, University of Copenhagen; ZSM, SNSB-Bavarian State Collection of Zoology, Munich.

Lineage	Tribe/group	Species	Status	Quant.	Catalog No.	Source	N	SL	Locality
***Gobius***	Benthophilini/ benthophilin	*Anatirostrum profundorum* (Berg, 1927)	CE	L, T	CMNFI 1999–0023.1	[45]	1	–	Southern Caspian Sea, Iran
		*Benthophiloides brauneri* Beling & Iljin, 1927	PCN	L, T	NMNH NASU 5136	[45]	1	50.0	Black Sea, Ukraine
		*Benthophilus abdurahmanovi* Ragimov, 1978	CE	L, T, G	ZMMU P.15890	[45]	1	33.0	Middle Caspian Sea, Russia
		*Benthophilus baeri* Kessler, 1877	CE	L, T, G	ZMMU P.16141	[45]	1	42.0	Caspian Sea, Turkmenistan
		*Benthophilus durrelli* Boldyrev & Bogutskaya, 2004	AZE	L, T, G	ZMMU P.21611	[45]	1	53.0	Azov Sea, Russia
		*Benthophilus leobergius* Berg, 1949	CE	L, T, G	ZM-CBSU 50–54; ZMMU P.22625	TS; [45]	5; 1	40.1–62.0; 56.0	Southern Caspian Sea, Iran; Northern Caspian Sea, Russia
		*Benthophilus macrocephalus* (Pallas, 1787)	CE	L, T, G	ZMMU P.15889	[45]	1	38.0	Middle Caspian Sea, Russia
		*Benthophilus pinchuki* Ragimov, 1982	CE	L, T, G	ZM-CBSU 119; ZMMU P.16127	TS; [45]	1; 1	61.1; 60.0	Southern Caspian Sea, Iran; Caspian Sea, Turkmenistan
		*Benthophilus stellatus* (Sauvage, 1874)	AZE	L, T, G	ZMMU P.11023	[45]	1	100.0	Sea of Azov
		*Caspiosoma caspium* (Kessler, 1877)	PCN	L, T	ZMMU P.13965	[12]	1	28.5	Ponto-Caspian basin
	Neogobiini/ neogobiin	*Neogobius bathybius* (Kessler, 1877)	CE	L, T, G, S	ZM-CBSU 101, 106, 108, 110–111	TS	5	210.0–221.0	Southern Caspian Sea, Iran
		*Neogobius caspius* (Eichwald, 1831)	CE	L, T, G, S	ZM-CBSU 2, 6, 9, 12–14	TS	6	54.7–93.2	Southern Caspian Sea, Iran
		*Neogobius fluviatilis* (Pallas, 1814)	BAN	L, T, G	ZMMU P.22433	[44]	1	81.0	Black Sea
		*Neogobius melanostomus* (Pallas, 1814)	PCN	L, T, G, S	ZM-CBSU 15–16, 18–19, 134; RBINS (uncat.)	TS; [43]	5; 1	47.9–75.3; 108.0	Southern Caspian Sea, Iran; Black Sea, Vienna
		*Neogobius pallasi* (Berg, 1916)	CE	L, T, G, S	ZM-CBSU 21–24, 27–28	TS	6	55.1–67.2	Southern Caspian Sea, Iran
	Ponticolini/ neogobiin	*Mesogobius batrachocephalus* (Pallas, 1814)	BAN	L, T	ZMUC P2395071-72	TS	2	–	Sea of Azov: Primorsk
		*Mesogobius nonultimus* (Iljin, 1936)	CE	L, T	ZM-CBSU S036-1	TS	1	126	Southern Caspian Sea, Iran
		*Ponticola gorlap* (Iljin, 1949)	CE	L, T, G, S	ZM-CBSU 41, 44–46, 48	TS	5	70.3–94.0	Southern Caspian Sea basin, Iran
		*Ponticola hircaniaensis* Zarei et al., 2022	CE	L, T, G	ZM-CBSU K18, K21, K24, K28	TS	4	64.0–74.6	Southern Caspian Sea basin, Iran
		*Ponticola iljini* (Vasil’eva & Vasil’ev, 1996)	CE	L, T, G	ZMMSU P-23516, P-23518	[38]	3	61.0–95.0	Middle Caspian Sea, Kazakhstan
		*Ponticola iranicus* Vasil’eva et al., 2015	CE	L, T, G, S	ZM-CBSU 37–40, 141, 144	TS	6	55.3–92.5	Southern Caspian Sea basin, Iran
		*Ponticola kessleri* (Günther, 1861)	BE	L, T, G	RBINS (uncat.); ZMMSU P-22456	[38, 43]	2; 1	77.0–92.0; 56.0	Black Sea basin, Vienna; Black Sea, Romania
		*Ponticola patimari* Eagderi et al., 2020	CE	L, T, G, S	ZM-CBSU P1–P4, P6	TS	5	51.7–65.6	Southern Caspian Sea basin, Iran
		*Ponticola syrman* (Nordmann, 1840)	PCN	L, T, G	ZM-CBSU 77–78	TS	2	128.1–181.4	Southern Caspian Sea, Iran
		*Proterorhinus nasalis* (De Filippi, 1863)	CE	L, T, G, S	ZM-CBSU 55–57, 59–62	TS	7	30.3–48.0	Southern Caspian Sea, Iran
		*Proterorhinus semilunaris* (Heckel, 1837)	BAN	–	ZSM Pis-041810, Pis-026426	TS	6*	39.0–53.3	Black Sea basin, Germany & Türkiye
***Pomatoschistus***	–	*Hyrcanogobius bergi* Iljin, 1928	CE	L, T, G, S	ZM-CBSU 83–91	TS	9	23.4–26.0	Southern Caspian Sea, Iran
		*Knipowitschia caucasica* (Berg, 1916)	PCN	L, T, G, S	ZM-CBSU 63–64, 80–82, 128–129	TS	7	29.5–36.0	Southern Caspian Sea, Iran
		*Knipowitschia longecaudata* (Kessler, 1877)	PCN	L, T, G, S	ZM-CBSU 65–69, 92–97	TS	11	20.0–29.7	Southern Caspian Sea, Iran
***Acanthogobius***	–	*Rhinogobius* sp.	EX	L, T, G, S	ZM-CBSU 70–76	TS	7	21.6–36.0	Southern Caspian Sea basin, Iran

*Otolith’s medial face damaged. TS: this study.

### Inclusivity in global research

Additional information regarding the ethical, cultural, and scientific considerations specific to inclusivity in global research is included in the Supporting Information (S1 Checklist).

### Otolith terminology, measurements, and variables

**Fig 1:**
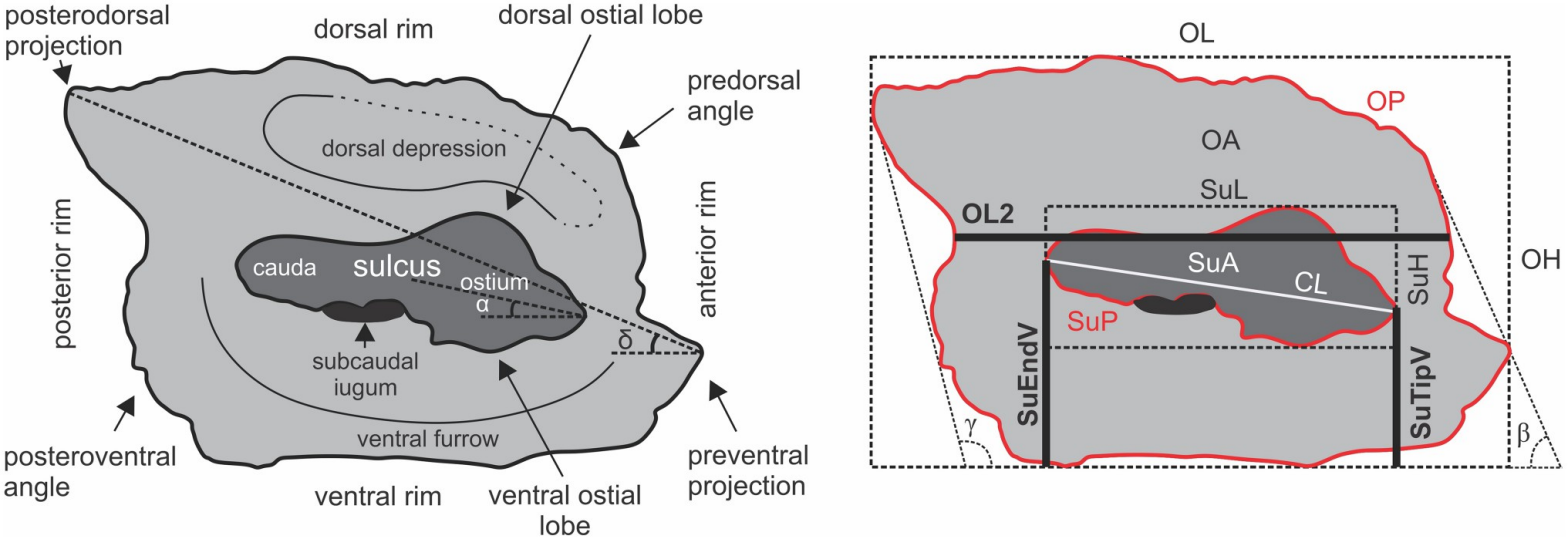
Schematic illustration of the left sagittal otolith medial face of *Ponticola gorlap*, showing the terminology for characters and morphometric measurements. OL, maximal otolith length; OL2, minimal otolith length; OH, maximal otolith height; OA, otolith area; OP, otolith perimeter; CL, colliculum length; SuL, sulcus length; SuH, sulcus height; SuP, sulcus perimeter; SuA, sulcus area; SuTipV, distance from sulcus tip to the ventral margin; SuEndV, distance from sulcus end to the ventral margin; α, inclination angle of ostium; β, inclination angle of anterior rim; γ, inclination angle of posterior rim; δ, angle of preventral projection to posterodorsal projection traverse.

Terminology for the otolith morphology (Fig 1) followed Gierl et al. [46] and Schwarzhans et al. [45]. Using the SEM images, 12 measurements were taken for each otolith in ImageJ 1.52a: OL, maximal otolith length; OL2, minimal otolith length; OH, maximal otolith height; CL, colliculum length; SuL, sulcus length; SuH, sulcus height; OP, otolith perimeter (in mm); OA, otolith area (in mm^2^); SuP, sulcus perimeter (in mm); SuA, sulcus area (in mm^2^); SuTipV and SuEndV, distance from sulcus tip and end to the ventral margin, respectively. These measurements were used to calculate 24 otolith variables [45, 46]: OL/OH (= aspect ratio or ASr), OP/OL, OP/OH, SuA/OA, SuP/OP, SuP/SuTipV, SuP/SuEndV, SuL/OL, SuL/OH, SuL/SuH, SuL/SuTipV, SuL/SuEndV, SuL/OP, SuL/SuP, SuH/OL, SuH/OH, SuH/SuTipV, SuH/SuEndV, SuH/OP, SuH/SuP, SuTipV/OP, SuTipV/SuEndV, SuEndV/OP, and OL2/CL. Four inclination angles were measured [45]: α, inclination angle of ostium; β, inclination angle of anterior rim; γ, inclination angle of posterior rim; δ, inclination of line connecting preventral angle with tip of posterodorsal projection. In addition to ASr [47], another three shape indices were calculated [47]: (i) roundness (ROx = 4OA/πOL^2^), which is larger when the shape is more circular; (ii) rectangularity [REx = OA/(OL×OH)], with 1 being a perfect square and <1 being a nonsquare; and (iii) ellipticity [ELx = (OL–OH)/(OL+OH)], ranging from zero (a perfect round shape) to close to 1.0 (a spindle shape).

### Otolith data analysis

The otoliths were analysed based on two different approaches:

(i) A descriptive method for all 30 species.
(ii) A quantitative method based on otolith morphometric variables and shape descriptors for which comparisons were made at four levels: lineage, tribe, genus, and species (Table 1). In the genus- and species-level analyses, genera and species with less than five otoliths were not considered. All otolith variables including morphometric ratios, inclination angles, and shape indices were analysed using IBM SPSS Statistics 26.0 [48]. The normal distributions of otolith variables for each species, genus, tribe, and lineage were compared using Shapiro-Wilk tests (p > 0.05). Mann-Whitney tests (p < 0.05) were used to evaluate the significance of non-normally distributed otolith variables; Univariate Analysis of Variance (ANOVA) with Tukey’s HSD (p < 0.05) and Dunnett’s T3 (p < 0.05) post-hoc tests (depending on homogeneity of variances, Levene’s test, p > 0.05) were used for taxon comparisons of normally distributed otolith variables. A Bonferroni correction (0.05/number of tests) was used at each taxonomic level to correct for multiple tests. Discriminant function analysis (DFA) was conducted to determine the proportion of otoliths that could be correctly assigned to their corresponding species, genera, tribes, and lineages. The classification success was tested by leave-one-out cross validation. A dendrogram was constructed based on Euclidean distance as a measure of dissimilarity to show the phenotypic relationships among the species. The between groups linkage method was used as the clustering algorithm.

### Molecular phylogeny

There have been a few efforts to assess molecular phylogenetic relationships among benthophilines [29, 30, 61]. Neilson & Stepien [29] were the first to estimate relationships within this group; they used a combined dataset of two mitochondrial (*cyt* b and COI) and two nuclear loci (RAG1 and S7) with maximum parsimony (MP), maximum likelihood (ML), and Bayesian inference (BI) approaches. They introduced a revised classification and nomenclature that does not, however, aptly fit with the present understanding of gobioid family and subfamily phylogenetics (e.g., presented in Agorreta et al. [28] and McCraney et al. [60]), however, their primary phylogenetic inferences and tribe designations remain helpful as they are not in disagreement with any familial and subfamilial systematics. However, the most comprehensive phylogenetic analysis of the benthophiline gobies in terms of specimens and species numbers and also geographic coverage was conducted by Zarei et al. [30] based on mitochondrial COI sequences using ML and BI approaches, on which the current classification was based. That mitochondrial phylogeny is similar to the combined mito-nuclear phylogeny of Neilson and Stepien [29], except for (i) slightly different placement of *Babka*; and (ii) with regard to the sister group relationship of Ponticolini with Neogobiini rather than with Benthophilini; that grouping was, however, not robustly supported in Neilson and Stepien [29].

## Results

### Otolith descriptions

#### *Gobius* lineage

**Fig 2:**
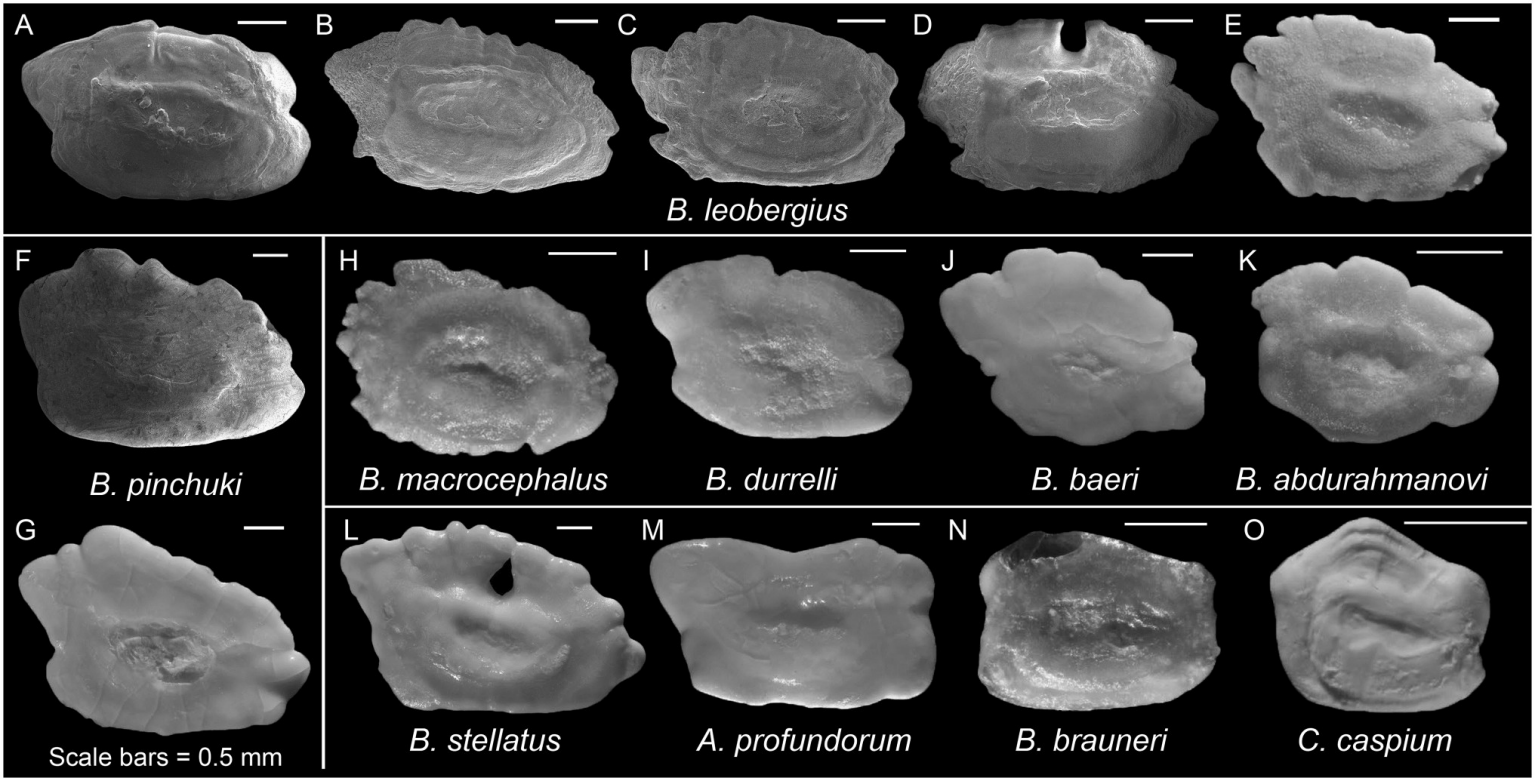
Otolith morphology of Benthophilini. *Benthophilus leobergius*: A, ZM-CBSU 50; B, ZM-CBSU 51; C, ZM-CBSU 53; D, ZM-CBSU 54; E, ZMMU P.22625. *Benthophilus pinchuki*: F, ZM-CBSU 119; G, ZMMU P.16127. *Benthophilus macrocephalus*: H, ZMMU P.15889. *Benthophilus durrelli*: I, ZMMU P.21611. *Benthophilus baeri*: J, ZMMU P.16141. *Benthophilus abdurahmanovi*: K, ZMMU P.15890. *Benthophilus stellatus*: L, ZMMU P.11023. *Anatirostrum profundorum*: M, CMNFI 1999–0023.1. *Benthophiloides brauneri*: N, NMNH NASU 5136. *Caspiosoma caspium*: O, ZMMU P.13965. Scale bars = 0.5 mm.

*Benthophilini*. ***Anatirostrum*. *Anatirostrum profundorum***. Right trapezoid (Fig 2M, from Schwarzhans et al. [45]); OL/OH 1.7; dorsal rim longer than ventral rim, with a shallow broad concavity in the middle, entire; predorsal angle orthogonal; posterodorsal projection long, broad, and rounded at tip; anterior rim vertical, slightly incised at the level of ostium, β 90°; posterior rim oblique, γ 111.3°, with a tiny incision slightly below the level of cauda; δ 22.1°; ventral rim horizontal, broadly undulate; preventral projection absent, orthogonal angle; posteroventral angle obtuse, nearly entire; sulcus centrally positioned, sole-shaped, horizontal, shallow, ostial lobes weakly developed; OL2/CL 2.7; subcaudal iugum indistinct; ventral furrow runs with a moderate distance to ventral rim; dorsal depression indistinct.

***Benthophilus*. *Benthophilus leobergius***. Long elliptical (Fig 2A–2E); OL/OH 1.6–1.7; dorsal rim regularly curved and broad, crenate or crenulate, highest behind mid-length above CA apex, anteriorly depressed; predorsal angle variable in size, pointed or rounded at tip; posterodorsal projection broad and long, pointed or rounded at tip, moderately bent outwards; anterior rim oblique, β 67.8–79.6°, variably incised at or above the level of ostium; posterior rim almost parallel to anterior rim, γ 109.4–116.0°, without or with a narrow deep incision below the level of CA; δ 10.8–13.2°; ventral rim horizontal, curved upwards anteriorly and posteriorly, entire or slightly crenulate; preventral projection short or long, rounded or pointed at tip; posteroventral angle usually acute and projected, sometimes almost orthogonal; sulcus centrally positioned, sole-shaped, horizontal, shallow, dorsal ostial lobe weakly developed, ventral ostial lobe developed; OL2/CL 1.8–2.1; subcaudal iugum indistinct; ventral furrow runs close to ventral rim; dorsal depression distinct and narrow.

***Benthophilus pinchuki***. Long elliptical (Fig 2F and 2G); OL/OH 1.4–1.4; dorsal rim broad and curved, highest behind mid-length above caudal apex, irregularly sinuate or crenate, anteriorly strongly depressed; predorsal angle indistinct; posterodorsal projection broad and long, rounded at tip, moderately bent outwards; anterior rim in continuation of dorsal rim, oblique, β 60.5–71.6°, slightly incised at the level of ostium; posterior rim less oblique comparing to the anterior rim, γ 104.4–108.2, with a small incision below the level of cauda; δ 16.9–17.0°; ventral rim horizontal, entire; preventral projection short, rounded or pointed at tip; posteroventral angle projected or orthogonal, entire; sulcus centrally positioned, sole-shaped, horizontal, shallow, ostial lobes weakly developed; OL2/CL 2.7–2.9; subcaudal iugum absent; ventral furrow runs with a moderate distance to ventral rim; dorsal depression indistinct or distinct and relatively wide.

***Benthophilus stellatus***. Long elliptical (Fig 2L, from Schwarzhans et al. [45]); OL/OH 1.6; dorsal rim broad and curved, highest behind mid-length above caudal apex, sinuate or crenate, anteriorly strongly depressed; predorsal angle and anterior rim in continuation of dorsal rim; posterodorsal projection broad and long, tapering at tip; anterior rim in continuation of dorsal rim, β 62.3°; posterior rim less oblique comparing to the anterior rim, γ 109.9, incision indistinct; δ 18.0°; ventral rim horizontal, entire, preventral projection moderately long, rounded at tip; posteroventral angle obtuse, slightly undulate; sulcus centrally positioned, sole-shaped, α 5.2°, shallow, ostial lobes weakly developed; OL2/CL 3.0; subcaudal iugum absent; ventral furrow runs with a moderate distance to ventral rim; dorsal depression distinct and relatively wide.

***Benthophilus baeri***. Long elliptical (Fig 2J, from Schwarzhans et al. [45]); OL/OH 1.3; dorsal rim broad, regularly curved, sinuate and crenate, highest behind mid-length behind caudal apex, anteriorly strongly depressed; predorsal angle and anterior rim in continuation of dorsal rim; posterodorsal projection broad and long, tapering at tip; anterior rim in continuation of dorsal rim, β 78.7°, incised at or slightly above the level of ostium; posterior rim oblique as anterior rim, γ 121.0°, incised slightly below the level of cauda; δ 23.6°; ventral rim almost horizontal, undulate; preventral projection short and broad; posteroventral angle undulate; sulcus small, centrally positioned, sole-shaped, α 14.0°, shallow, ostial lobes weakly developed; OL2/CL 2.9; subcaudal iugum absent; ventral furrow runs with a moderate distance to ventral rim; dorsal depression distinct and relatively wide.

***Benthophilus abdurahmanovi***. Long elliptical (Fig 2K, from Schwarzhans et al. [45]); OL/OH 1.3; dorsal rim broadly crenate, highest behind mid-length above cauda, anteriorly strongly depressed; predorsal angle well-developed and rounded; posterodorsal projection very short, blunt; anterior rim almost vertical or little oblique, β 83.5°, incised above the level of ostium; posterior rim almost vertical, γ 95.9°, with an incision above the level of cauda; δ 20.7°; ventral rim curved, entire or broadly crenate; preventral projection short but broad, rounded; posteroventral angle markedly broad, obtuse, slightly undulate; sulcus centrally positioned, sole-shaped, α 13.0°, shallow, ostial lobes weakly developed; OL2/CL 2.8; subcaudal iugum absent; ventral furrow runs with a close distance to ventral rim; dorsal depression distinct and relatively narrow.

***Benthophilus durrelli***. Long elliptical (Fig 2I, from Schwarzhans et al. [45]); OL/OH 1.4; dorsal rim gently curved, mostly entire with a shallow incision in mid-length, highest behind incision above cauda, anteriorly slightly depressed; predorsal angle well-developed, broad and round; posterodorsal projection broad and long, rounded at tip; anterior rim little oblique, β 82.9°, incised slightly above the level of ostium; posterior rim more oblique comparing to the anterior rim, γ 108.1°, with a broad incision at the level of cauda; δ 19.1; ventral rim nearly horizontal or gently curving, entire; preventral projection short and broad, rounded at tip; posteroventral angle slightly projected, entire; sulcus centrally positioned, sole-shaped, horizontal, shallow, ostial lobes weakly developed; OL2/CL 2.4; subcaudal iugum absent; ventral furrow runs with a moderate distance to ventral rim; dorsal depression distinct and relatively narrow.

***Benthophilus macrocephalus***. Long elliptical (Fig 2H, from Schwarzhans et al. [45]); OL/OH 1.4; dorsal rim curved, highest behind caudal apex, irregularly crenate and sinuate, anteriorly strongly depressed; predorsal angle and anterior rim in continuation of dorsal rim; posterodorsal projection short and broad, rounded but strongly crenulate; anterior rim crenulate, incision not recognizable, oblique, β 63.4°; posterior rim crenulate, less oblique comparing to the anterior rim, γ 104.3°, incision not recognizable; δ 13.1°; ventral rim nearly horizontal or gently curving, crenulate; preventral projection short and rounded at tip; posteroventral angle crenulate; sulcus centrally positioned, sole-shaped, α 13.7°, shallow, dorsal ostial lobe indistinct, ventral ostial lobe weakly developed; OL2/CL 2.4; subcaudal iugum absent; ventral furrow runs with a close to moderate distance to ventral rim; dorsal depression distinct and relatively narrow.

***Benthophiloides*. *Benthophiloides brauneri***. Long rectangle (Fig 2N, from Schwarzhans et al. [45]); OL/OH 1.4; dorsal rim entire, posterior part horizontal to convex and regularly curved, highest above cauda; predorsal angle nearly orthogonal; posterodorsal projection very short and rounded; anterior rim vertical, β 90°, not incised; posterior rim almost vertical, γ ~90°, with a shallow broad concavity at the level of cauda; δ 22.0°; ventral rim horizontal, entire; preventral projection absent, almost orthogonal angle; posteroventral angle orthogonal, entire; sulcus centrally positioned, sole-shaped, horizontal, shallow, ostial lobes weakly developed; OL2/CL 1.8; subcaudal iugum absent; ventral furrow runs with a moderate distance to ventral rim; dorsal depression distinct and narrow.

***Caspiosoma*. *Caspiosoma caspium***. Pentagonal (Fig 2O, from Schwarzhans et al. [12]); OL/OH 1.1; dorsal rim angular, highest above cauda, entire; predorsal angle obtuse; posterodorsal projection very short or indistinct, obtuse angle; anterior rim incised slightly below the level of ostium, vertical, β ~90°; posterior rim not incised, γ 97.4°; δ 24.3°; ventral rim horizontal, entire; preventral projection very short, tapering at tip; posteroventral angle almost orthogonal or slightly obtuse, entire; sulcus centrally positioned, sole-shaped, α 7.5°, relatively deep, dorsal ostial lobe indistinct, ventral ostial lobe developed; OL2/CL 2.0; subcaudal iugum absent; ventral furrow runs with a close distance to ventral rim; dorsal depression distinct.

**Fig 3:**
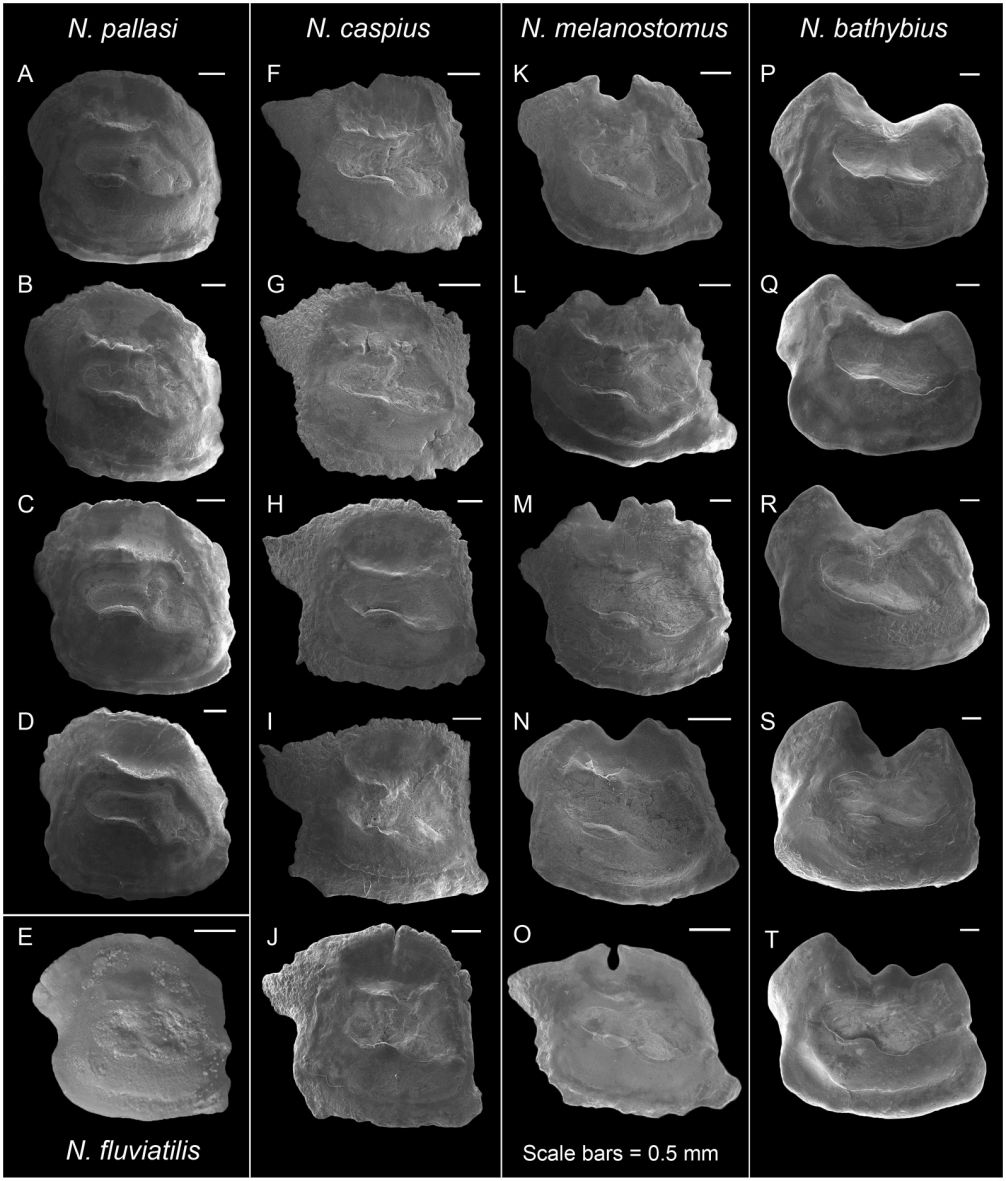
Otolith morphology of Neogobiini. *Neogobius pallasi*: A, ZM-CBSU 20; B, ZM-CBSU 22; C, ZM-CBSU 23; D, ZM-CBSU 27. *Neogobius fluviatilis*: E, ZMMU, P.22433. *Neogobius caspius*: F, ZM-CBSU 2; G, ZM-CBSU 6; H, ZM-CBSU 12; I, ZM-CBSU 13; J, ZM-CBSU 14. *Neogobius melanostomus*: K, ZM-CBSU 15; L, ZM-CBSU 16; M, ZM-CBSU 18; N, ZM-CBSU 19; O, unnumbered RBINS specimen [43]. *Neogobius bathybius*: P, ZM-CBSU 101; Q, ZM-CBSU 106; R, ZM-CBSU 108; S, ZM-CBSU 111; T, ZM-CBSU 110. Scale bars = 0.5 mm.

*Neogobiini*. ***Neogobius*. *Neogobius bathybius***. Two-humped long rectangle (Fig 3P–3T); OL/OH 1.1–1.2; dorsal rim two-humped with a deep broad V-shaped concavity at mid-length (i.e., M-shaped dorsal rim), humps angular, posterior hump markedly broader and higher, outline entire; posterodorsal projection bulky and very broad, not bending outwards; anterior rim usually little oblique, β 77.3–84.7°, with or without incision, entire; posterior rim almost parallel to the anterior rim or slightly more oblique, γ 96.7–105.9°, usually with a shallow broad concavity; δ 13.5–22.1°; ventral rim almost horizontal or gently curving, entire; preventral projection usually absent or a short round projection present; posteroventral angle almost orthogonal, entire; sulcus supramedian, sole- or dumbbell-shaped, cauda almost as wide as ostium, α 2.1–10.6°, very deep, ostial lobes usually well-developed; OL2/CL 1.5–1.6; subcaudal iugum usually absent, its length 1/2 cauda length and very slender if present; ventral furrow runs with a large distance to ventral rim; dorsal field absent.

***Neogobius caspius***. Square-rhomboid (Fig 3F–3J); OL/OH 1.1–1.3; dorsal rim curved, highest at mid-length or in front of it above ostium, posteriorly strongly depressed, dentate, denticulate or crenulate; predorsal angle almost orthogonal to slightly obtuse; posterodorsal projection very long, narrow, tapering and pointed, strongly bent outwards and dorsally as well; anterior rim vertical or little oblique, β 77.0–85.7°, not incised, dentate, denticulate or entire, higher than posterior rim; posterior rim oblique as anterior rim, γ 102.4–111.6°, with or without a shallow concavity below the projection; δ 26.6–33.4°; ventral rim nearly horizontal, sinuate or crenulate; preventral projection short and usually pointed; posteroventral angle orthogonal, entire or slightly denticulate/crenulate; sulcus centrally positioned, sole-shaped, α 14.8–17.9°, relatively deep, ostial lobes developed; OL2/CL 1.4–1.8; subcaudal iugum present, 1/2 cauda length, very slender; ventral furrow runs with a moderate distance to ventral rim; dorsal depression distinct and wide.

***Neogobius fluviatilis***. Discoid-rhomboid (Fig 3E, from Bratishko et al. [44]); OL/OH 1.1; dorsal rim convex, regularly curved, slightly highest behind mid-length above cauda, entire to slightly crenate, anteriorly depressed; predorsal angle obtuse; posterodorsal projection long and broad, slightly tapering or blunt at tip; anterior rim almost vertical or little oblique, β 85.6°, incision not recognizable from crenation; posterior rim more oblique comparing to the anterior rim, γ 104.5°, a broad concavity presents below the projection; δ 24.7°; ventral rim nearly horizontal, entire to slightly sinuate; preventral projection not recognizable from crenation; posteroventral angle nearly orthogonal, entire; sulcus centrally positioned, sole-shaped, α 14.3°, shallow, ostial lobes weakly developed; OL2/CL 1.6; subcaudal iugum present, 1/2 cauda length, relatively narrow; ventral furrow runs with a moderate distance to ventral rim; dorsal depression distinct and wide.

***Neogobius melanostomus***. Square-rhomboid (Fig 3K–3O); OL/OH 1.1–1.2; dorsal rim convex, regularly curved, crenate, coarsely crenate or entire, with a deep U-shaped indentation at mid-length; predorsal angle slightly obtuse to orthogonal; posterodorsal projection long and tapering, usually blunt at tip, strongly bent outwards; anterior rim oblique, β 77.3–84.6°, incision absent or not recognizable from dentation; posterior rim oblique as anterior rim, γ 102.3–109.4°, usually without or rarely with a shallow concavity; δ 24.7–27.2°; ventral rim nearly horizontal or gently curved, entire or undulate; preventral projection long, and blunt or pointed at tip; posteroventral angle nearly orthogonal, usually entire, sometimes dentate; sulcus centrally positioned, sole-shaped, α 17.0–19.8°, relatively deep, ostial lobes well-developed; OL2/CL 1.4–1.7; subcaudal iugum 1/2 cauda length, thick; ventral furrow runs with a moderate distance to ventral rim; dorsal depression distinct and relatively wide.

***Neogobius pallasi***. Discoid-rhomboid (Fig 3A–3D); OL/OH 1.0–1.1; dorsal rim convex, regularly curved, highest behind mid-length above cauda, entire to denticulate, anteriorly depressed; predorsal angle obtuse or in continuation of dorsal rim; posterodorsal projection short and broad, rounded or blunt at tip, slightly bent outwards; anterior rim nearly vertical or slightly oblique, β 83.9–88.8°, incision not recognizable from crenation; posterior rim little more oblique comparing to the anterior rim, γ 95.7–112.2°, usually with a shallow concavity; δ 24.3–31.0°; ventral rim nearly horizontal to gently curved, entire to slightly undulate; preventral projection absent; posteroventral angle nearly orthogonal, entire to slightly undulate; sulcus centrally positioned, sole-shaped, α 16.0–22.5°, relatively deep, ostial lobes well-developed; OL2/CL 1.4–1.6; subcaudal iugum 1/2 cauda length, thick to relatively slender; ventral furrow runs with a moderate to close distance to ventral rim; dorsal depression distinct and wide.

**Fig 4:**
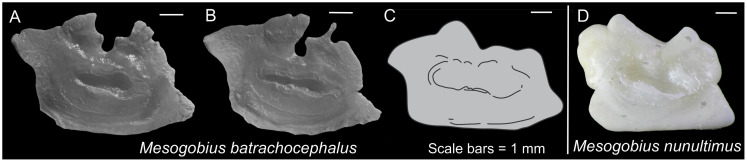
Otolith morphology of *Mesogobius*. *Mesogobius batrachocephalus*: A–B, ZMUC P2395071-72; C, drawing from Nolf [49]. *Mesogobius nonultimus*: D, ZM-CBSU S036-1. Scale bars = 1 mm.

*Ponticolini*. ***Mesogobius*. *Mesogobius batrachocephalus***. Long parallelogram with a deep irregular dorsal concavity (Fig 4A–4C); OL/OH 1.5–1.6; dorsal rim with a deep irregular concavity at mid-length, highest behind mid-length; posterodorsal projection very long and pointed, slightly bending outwards; anterior rim oblique, β 60.6–67.1°, slightly incised at the level of ostium, slightly dentate to sinuate; posterior rim parallel to the anterior rim, γ 112.5–113.1°, not incised; δ 15.1–21.8°; ventral rim almost horizontal, gently curving, entire to slightly undulate; preventral projection very long and pointed; posteroventral angle orthogonal to obtuse, entire to slightly undulate; sulcus centrally positioned, sole-shaped, α 6.8–13.4°, relatively deep, ostial lobes developed; OL2/CL 1.4–1.6; subcaudal iugum present, its length 1/2 cauda length and slender; ventral furrow runs with a close to intermediate distance to ventral rim; dorsal depression distinct and wide.

***Mesogobius nunultimus***. Two-humped long rectangle (Fig 4D); OL/OH 1.3; dorsal rim two-humped with a deep broad V-shaped concavity at mid-length (i.e., M-shaped dorsal rim), humps angular, posterior hump markedly broader and higher, outline entire to sinuate; posterodorsal projection bulky and very broad, not bending outwards; anterior rim oblique, β 73.7°, with a small incision above the level of ostial apex, entire; posterior rim almost parallel to the anterior rim or slightly less oblique, γ 101.6°, with a shallow small incision below the level of cauda; δ 27.2°; ventral rim almost horizontal, entire; preventral projection very long and pointed; posteroventral angle rounded, entire; sulcus centrally positioned, sole-shaped, α 17.5°, relatively deep, ostial lobes developed; OL2/CL 1.7; subcaudal iugum present, its length 1/2 cauda length and slender; ventral furrow runs with a moderate to large distance to ventral rim; dorsal depression indistinct.

**Fig 5:**
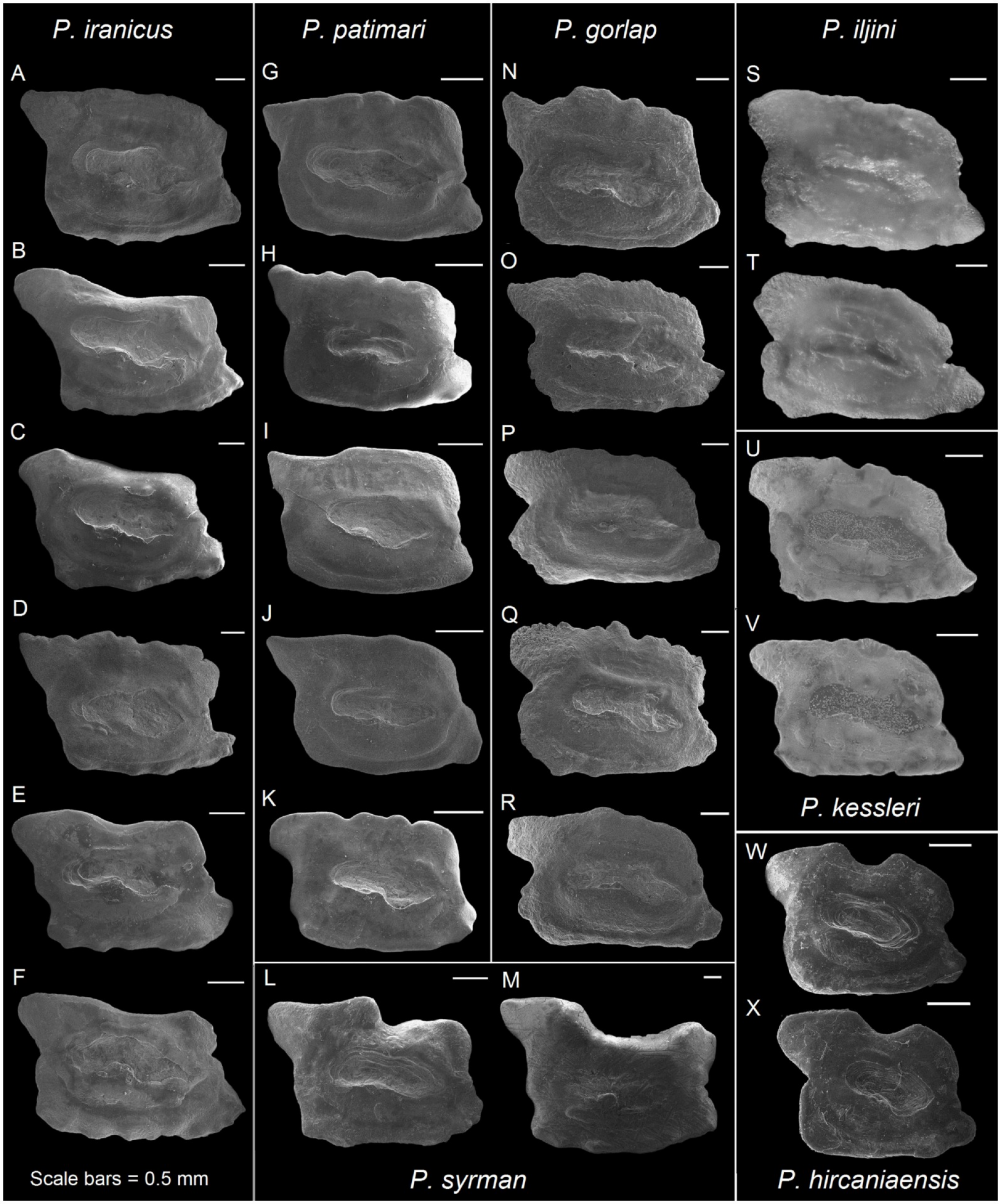
Otolith morphology of *Ponticola*. *Ponticola iranicus*: A, ZM-CBSU 37; B, ZM-CBSU 38; C, ZM-CBSU 39; D, ZM-CBSU 40; E, ZM-CBSU 141; F, ZM-CBSU 144. *Ponticola patimari*: G, ZM-CBSU P1; H, ZM-CBSU P2; I, ZM-CBSU P3; J, ZM-CBSU P4; K, ZM-CBSU P6. *Ponticola syrman*: L, ZM-CBSU 142; M, ZM-CBSU 139. *Ponticola gorlap*: N, ZM-CBSU 41; O, ZM-CBSU 44; P, ZM-CBSU 45; Q, ZM-CBSU 46; R, ZM-CBSU 48. *Ponticola iljini*: S–T, ZMMSU P-23516; U–V, *Ponticola kessleri*: non-catalogued specimens from the RBINS collection, Danube, near Vienna in Austria [43]. *Ponticola hircaniaensis*: W, ZM-CBSU K18; X, ZM-CBSU K28 [5]. Scale bars = 0.5 mm.

***Ponticola*. *Ponticola gorlap***. Long parallelogram (Fig 5N–5R); OL/OH 1.3–1.6; dorsal rim horizontal, irregularly sinuate and dentate, anteriorly slightly depressed; predorsal angle obtuse; posterodorsal projection moderately long and tapering, pointed or blunt at tip, strongly bent outwards; anterior rim oblique, β 66.1–81.3°, not incised or slightly incised at the level of ostium or above it; posterior rim almost parallel to the anterior rim or a little less oblique, γ 92.5–104.6°, with a marked concavity at or slightly above the level of cauda; δ 24–28°; ventral rim horizontal, entire or slightly undulate, slightly projecting downwards near the posterior end; preventral projection long, pointed or blunt at tip; posteroventral angle usually broadly rounded, undulate, crenate or denticulate; sulcus centrally positioned, sole-shaped, α 9.4–18.8°, moderately deep, ostial lobes well-developed, long, relatively wide, OL2/CL 1.4–1.5; subcaudal iugum present, 1/3 cauda length, slender; ventral furrow runs with a moderate to close distance to ventral rim; dorsal depression distinct, narrow to moderately wide.

***Ponticola iljini***. Long parallelogram (Fig 5S abd 5T, from Vasil’eva et al. [38]); OL/OH 1.5–1.8; dorsal rim nearly horizontal, anteriorly strongly depressed, entire to slightly undulate; predorsal angle obtuse; posterodorsal projection long and broad, tapering or blunt at tip, strongly bent outwards; anterior rim oblique, β 61.5–72.0°, incised at the level of ostium; posterior rim less oblique comparing to the anterior rim, γ 95.7–112.2°, incised at the level of cauda; δ 16.8–27.8°; ventral rim horizontal, undulate or sinuate, slightly projecting downwards near the posterior end; preventral projection long and tapering; posteroventral angle broadly rounded, crenate; sulcus centrally positioned, sole-shaped, α 13.7–15.4°, moderately deep, ostial lobes developed; OL2/CL 1.6–1.7; subcaudal iugum present, 1/3 cauda length, slender; ventral furrow runs with a moderate to close distance to ventral rim; dorsal depression distinct, narrow to moderately wide.

***Ponticola hircaniaensis***. Long parallelogram (Fig 5W and 5X); OL/OH 1.2–1.4; dorsal rim horizontal with a broad U-shaped concavity in the middle, entire; predorsal angle obtuse; posterodorsal projection highly positioned, long and broad, pointed or blunt at tip, slightly bents outwards; anterior rim oblique, β 72.3–84.3°, broadly incised at or slightly above the level of ostium; posterior rim almost parallel to the anterior rim, γ 101.3–104.9°, incised at or slightly below the level of cauda; δ 24.4–34.1°; ventral rim horizontal and entire; preventral projection usually long, sometimes short, pointed or blunt at tip; posteroventral angle usually orthogonal, entire or undulate; sulcus centrally positioned, sole-shaped, α 10.2–19.1°, very deep, with developed ostial lobes; OL2/CL 1.6–1.7; subcaudal iugum present, usually 1/3 cauda length, slender; ventral furrow runs with a moderate distance to ventral rim; dorsal depression indistinct.

***Ponticola iranicus***. Long parallelogram (Fig 5A–5F); OL/OH 1.4–1.6; dorsal rim nearly horizontal, sometimes with a shallow broad concavity in the middle, usually entire, sometimes crenate; predorsal angle slightly obtuse; posterodorsal projection very long and broad, pointed or blunt at tip, highly positioned, strongly bent outwards, and facing upwards dorsally; anterior rim oblique, β 66.1–78.9°, with a broad concavity or incision not recognizable from crenation; posterior rim almost parallel to the anterior rim, γ 103.7–110.9°, without a recognizable incision or notch; δ 20.9–28.6°; ventral rim horizontal, undulate or sinuate, usually slightly projecting downwards near the posterior end; preventral projection long, pointed or blunt at tip; posteroventral angle usually obtuse, sometimes orthogonal, entire, undulate or sinuate; sulcus centrally positioned, sole-shaped, α 11.0–16.4°, very deep, ostial lobes usually well-developed; OL2/CL 1.4–1.7; subcaudal iugum present or indistinct, 1/2 cauda length if present, slender; ventral furrow runs with a close to large distance to ventral rim; dorsal depression absent or distinct, narrow to moderately wide if distinct.

***Ponticola kessleri***. Long parallelogram (Fig 5U and 5V, from Vasil’eva et al. [38], and Jacobs and Hoedemakers [43]); OL/OH 1.3–1.6; dorsal rim nearly horizontal, anteriorly depressed, crenate, sinuate or undulate; predorsal angle obtuse; posterodorsal projection long and broad, blunt at tip, strongly bent outwards; anterior rim oblique, β 60.2–79.1°, slightly incised at the level of ostium; posterior rim less oblique comparing to the anterior rim, inclined at 95.4–108.0°, without or with a distinct incision; δ 24.1–28.3°; ventral rim horizontal, entire to slightly undulate, slightly projecting downwards near the posterior end; preventral projection long, and pointed or blunt at tip; posteroventral angle almost orthogonal or obtuse, crenate or sinuate; sulcus centrally positioned, sole-shaped, α 10.7–18.4°, moderately deep, ostial lobes developed; OL2/CL 1.4–1.5; subcaudal iugum present, 1/2 cauda length, slender; ventral furrow runs with a moderate to close distance to ventral rim; dorsal depression distinct or indistinct, narrow to moderately wide if distinct.

***Ponticola patimari***. Long parallelogram (Fig 5G–5K); OL/OH 1.4–1.6; dorsal rim horizontal, entire, crenate or sinuate; predorsal angle orthogonal to slightly obtuse; posterodorsal projection long, usually pointed or blunt at tip, strongly bent outwards and usually slightly facing upwards dorsally; anterior rim oblique, β 68.1–77.8°, with or without incision at the level of ostium; posterior rim parallel to the anterior rim, γ 98.5–109.5°, without or with a shallow concavity; δ 22.2–27.6°; ventral rim horizontal, usually entire to slightly undulate; preventral projection long, pointed or blunt at tip; posteroventral angle orthogonal, usually entire to slightly undulate; sulcus centrally positioned, sole-shaped, α 7.8–13.8°, very deep, ostial lobes developed; OL2/CL 1.5–1.8; subcaudal iugum usually absent or small, 1/2–1/3 cauda length, slender; ventral furrow runs with a moderate distance to ventral rim; dorsal depression usually distinct and moderately wide.

***Ponticola syrman***. Long parallelogram (Fig 5L and 5M); OL/OH 1.4; dorsal rim horizontal with a broad concavity in the middle, entire to slightly sinuate; predorsal angle well-developed, projecting forwards and upwards dorsally; posterodorsal projection very long and broad, highly positioned, tapering or pointed at tip, strongly bent outwards; anterior rim nearly vertical or little oblique, β 78.5–82.6°, with a broad concavity in the middle; posterior rim oblique, γ 105.4–108.0°, not incised; δ 23.5–27.3°; ventral rim nearly horizontal or gently curving, entire to slightly undulate; preventral projection short, blunt at tip; posteroventral angle nearly orthogonal or slightly obtuse, sometimes undulate or sinuate; sulcus centrally positioned, sole-shaped, α 4.7–13.3°, very deep, dorsal ostial lobe weakly developed or indistinct, ventral ostial lobe developed; OL2/CL 1.7–1.9; subcaudal iugum indistinct or very small, 1/4 cauda length; ventral furrow runs with a large distance to ventral rim; dorsal depression absent or indistinct.

**Fig 6:**
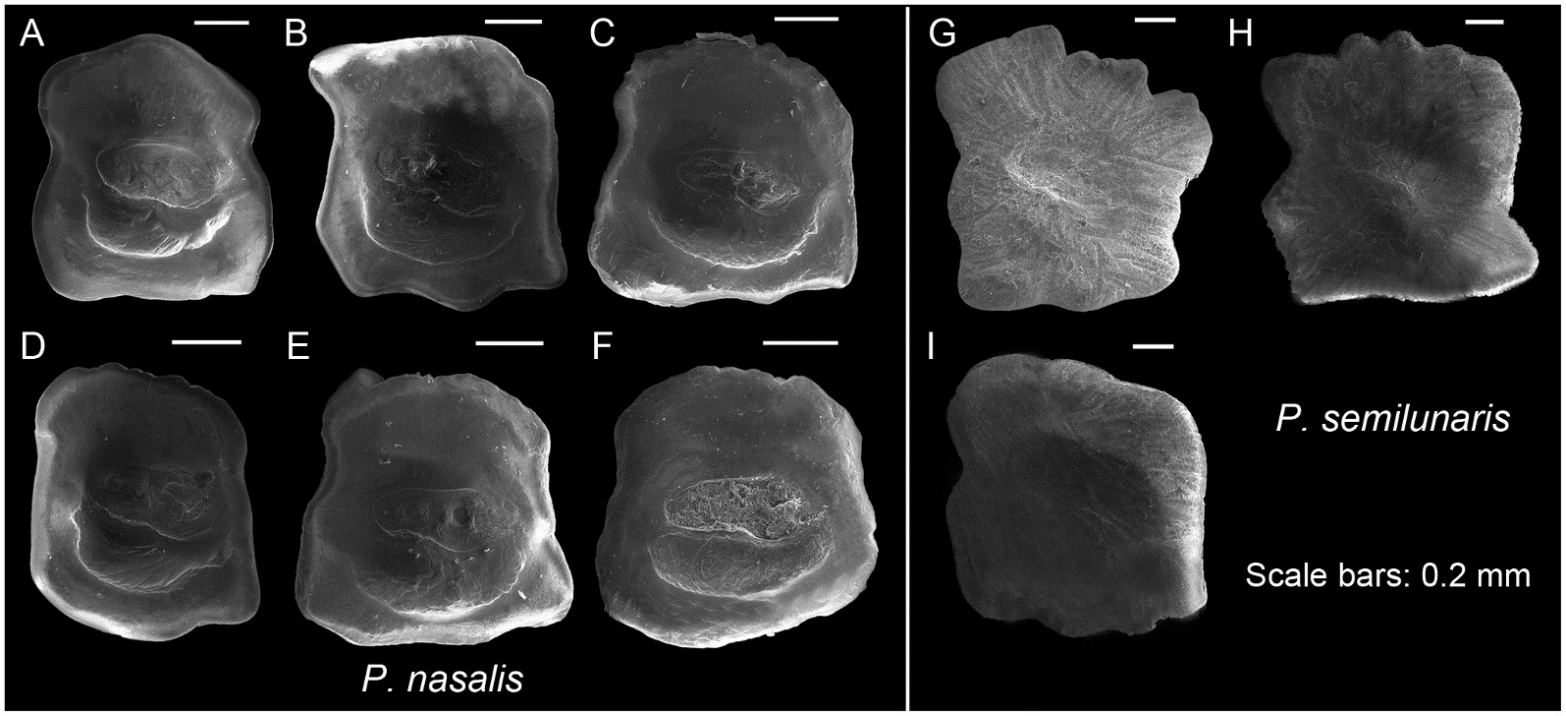
Otolith morphology of *Proterorhinus*. *Proterorhinus nasalis*: A, ZM-CBSU 56; B, ZM-CBSU 57; C, ZM-CBSU 59; D, ZM-CBSU 60; E, ZM-CBSU 61; F, ZM-CBSU 62. *Proterorhinus semilunaris*: G, ZM-CBSU 122; H, ZM-CBSU 126; I, ZM-CBSU 125. Scale bar = 0.2 mm.

***Proterorhinus*. *Proterorhinus nasalis***. Square-trapezoid (Fig 6A–6F); OL/OH 0.8–1.0; dorsal rim variable in shape, rounded, horizontal, and sometimes angular in middle, entire or crenulate, usually shorter than ventral rim; predorsal angle obtuse to orthogonal; posterodorsal projection very short; anterior rim nearly vertical to little oblique, β 81.0–88.4°, usually not incised or sometimes incised above the level of ostium; posterior rim oblique to vertical, γ 80.0–90.0°, usually with a shallow broad incision above the level of cauda; δ 34.2–41.4°; ventral rim horizontal, longer than dorsal rim, usually broadly undulate; preventral projection absent to short, rounded, pointed or blunt; posteroventral angle projected, broad and rounded, sometimes nearly orthogonal; sulcus centrally positioned, sole-shaped, α 0.0–14.8°, moderately deep, ostial lobes variably developed; OL2/CL 1.5–1.8; subcaudal iugum usually indistinct to very small, 1/3 cauda length; ventral furrow runs with a large distance to ventral rim; dorsal depression indistinct or distinct and wide.

***Proterorhinus semilunaris***. Square (Fig 6G–6I); OL/OH 0.9–1.0; dorsal rim variable in shape, horizontal or depressed anteriorly, undulate to crenate; predorsal angle obtuse to orthogonal; posterodorsal projection broad, rounded to blunt; anterior rim nearly vertical, β 82.1–108.2°, with or without a small incision; posterior rim nearly parallel to anterior rim, γ 79.1–90.0°, with a shallow broad incision at the level of cauda; δ 33.3–39.1°; ventral rim horizontal, broadly undulate; preventral projection absent, short or long, blunt or pointed; posteroventral angle nearly orthogonal or little obtuse, broadly undulated; sulcus centrally positioned, sole-shaped, α 11.1–20.2°, moderately deep to shallow, ostial lobes variably developed; OL2/CL 1.6–2.1; subcaudal iugum and ventral furrow indistinct because of bad preservation; dorsal depression indistinct or distinct and wide.

**Fig 7:**
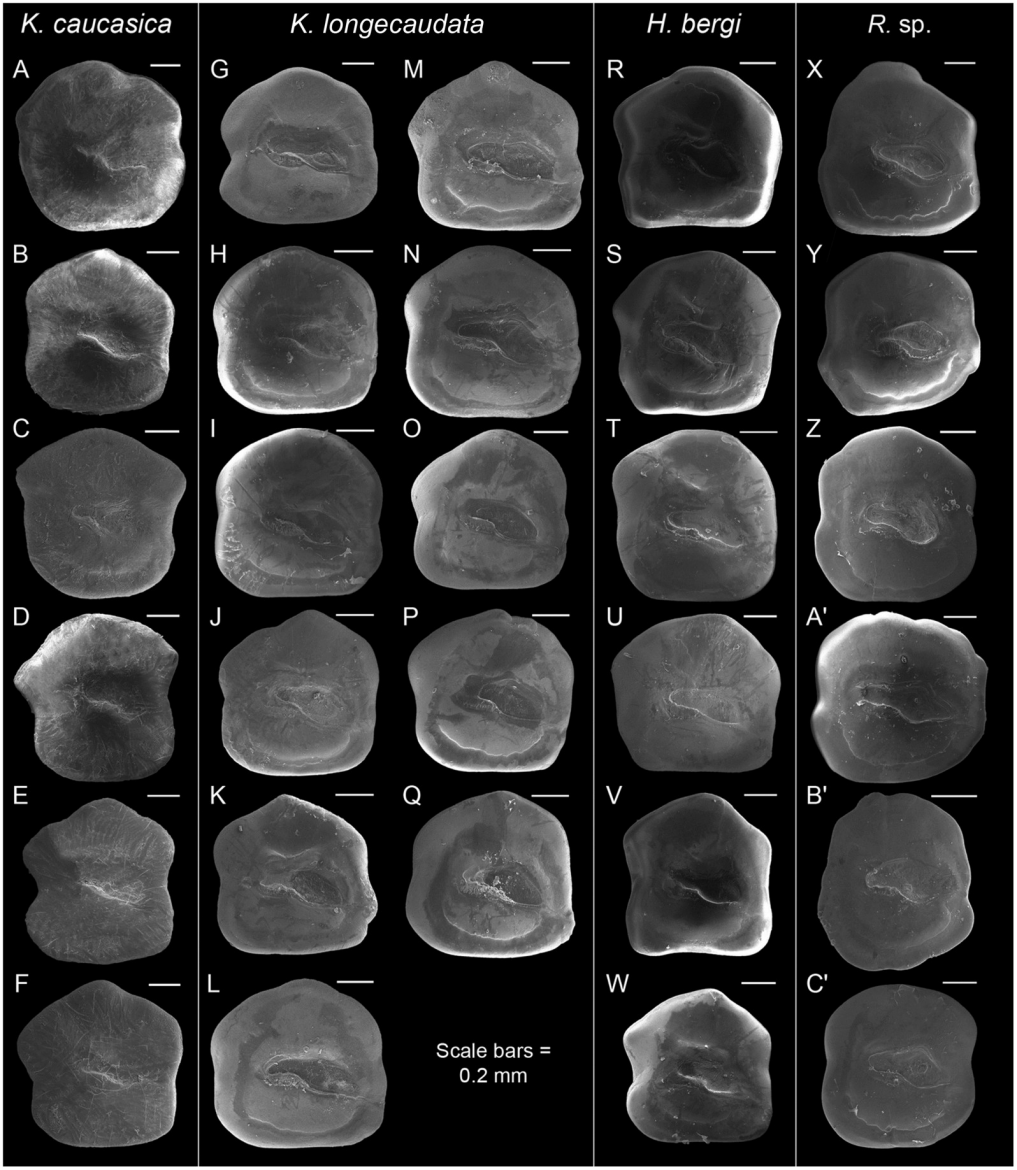
Otolith morphology of the studied species from the *Pomatoschistus* and *Acanthogobius* lineages. *Knipowitschia caucasica*: A, ZM-CBSU 63; B, ZM-CBSU 64; C, ZM-CBSU 80; D, ZM-CBSU 82; E, ZM-CBSU 128; F, ZM-CBSU 129. *Knipowitschia longecaudata*: G, ZM-CBSU 65; H, ZM-CBSU 66; I, ZM-CBSU 67; J, ZM-CBSU 68; K, ZM-CBSU 69; L, ZM-CBSU 92; M, ZM-CBSU 93; N, ZM-CBSU 94; O, ZM-CBSU 95; P, ZM-CBSU 96; Q, ZM-CBSU 97. *Hyrcanogobius bergi*: R, ZM-CBSU 84; S, ZM-CBSU 85; T, ZM-CBSU 86; U, ZM-CBSU 87; V, ZM-CBSU 90; W, ZM-CBSU 91. *Rhinogobius* sp.: X, ZM-CBSU 70; Y, ZM-CBSU 71; Z, ZM-CBSU 72; A’, ZM-CBSU 73; B’, ZM-CBSU 74; C’, ZM-CBSU 76. Scale bars = 0.2 mm.

***Pomatoschistus* lineage**. *Knipowitschia*. ***Knipowitschia caucasica***. Pentagonal (Fig 7A–7F); OL/OH 0.9–1.0; dorsal rim angular, highest medially, thereafter inclined with a steep slope anteriorly and posteriorly, usually entire; predorsal angle and posterodorsal projection usually distinct and somewhat symmetrical; otolith sometimes widest across predorsal angle to posterodorsal projection; anterior rim vertical to little oblique, β 90.0–97.6°, with a broad shallow incision at the level of ostium or slightly above it; posterior rim vertical to oblique, γ 90.0–110.1°, usually with a broad shallow incision at the level of cauda; δ 26.3–40.2°; ventral rim usually horizontal or little curved, entire; preventral projection absent; posteroventral angle nearly orthogonal, little obtuse or projected, entire; sulcus centrally positioned, sole-shaped, α 12.7–22.7°, moderately deep or shallow, ostial lobes variably developed; OL2/CL 1.8–2.2; subcaudal iugum indistinct (because of bad preservation); ventral furrow indistinct (because of bad preservation) or runs with a moderate distance to ventral rim; dorsal depression indistinct or distinct and wide.

***Knipowitschia longecaudata***. Pentagonal (Fig 7G–7Q); OL/OH 0.9–1.0; dorsal rim angular, highest infront of mid-length, thereafter inclined with a steep slope anteriorly and posteriorly, entire; predorsal angle and posterodorsal projection obtuse; anterior rim almost vertical, β 81.3–90.0, usually without or with a tiny shallow incision above the level of ostium; posterior rim nearly vertical, γ 87.4–104.7, with a broad shallow incision at the level of cauda; δ 24.9–30.4; ventral rim horizontal, entire; sulcus centrally positioned, sole-shaped, α 15.5–26.1, moderately deep, ostial lobes variably developed; OL2/CL 1.7–2.1; subcaudal iugum thick and long, as long as cauda; ventral furrow runs with a large to moderate distance to ventral rim; dorsal depression indistinct or distinct and wide.

*Hyrcanogobius*. ***Hyrcanogobius bergi***. Pentagonal (Fig 7R–7W); OL/OH 0.9–1.0; dorsal rim usually angular to rounded, highest medially, thereafter inclined with a steep slope anteriorly and posteriorely, entire; predorsal angle and posterodorsal projection obtuse; anterior rim nearly vertical, β 81.3–90.0°, usually without incision, sometimes a small shallow incision above level of ostium present; posterior rim nearly vertical, γ 87.4–104.7°, with a broad shallow incision at the level of cauda; δ 24.9–30.4°; ventral rim horizontal, entire, preventral projection absent; sulcus centrally positioned, sole-shaped, α 15.5–26.1°; moderately deep, ostial lobes variably developed; OL2/CL 1.7–2.1; subcaudal iugum large and long, as long as cauda or slightly shorter; ventral furrow runs with a moderate distance to ventral rim; dorsal depression indistinct or distinct and wide.

#### *Acanthogobius* lineage

*Rhinogobius*. ***Rhinogobius sp***. Almost pentagonal or with a rounded dorsal margin (Fig 7X–7C’); OL/OH 0.9–1.0; dorsal rim angular or rounded, highest medially, entire to sinuate; predorsal angle and posterodorsal projection obtuse; anterior rim nearly horizontal, β ~90°, without or with a small shallow incision; posterior rim nearly vertical, γ ~90°, with or without a shallow broad incision at the level of cauda; δ 32.6–42.5°; ventral rim usually horizontal, sometimes rounded, entire to little undulate; preventral projection usually indistinct to very small; sulcus centrally positioned, sole-shaped, α 12.9–24.1°, moderately deep, ostial lobes variably developed; OL2/CL 1.6–2.2; subcaudal iugum wide and long, as long as cauda or slightly shorter; ventral furrow runs with a moderate distance to ventral rim; dorsal depression indistinct.

### Otolith morphometric variables and classical shape descriptors

#### Variation among the lineages

Twenty-eight otolith variables significantly differed between two or more of the three lineages (Table 2, S1 Fig, S1 Table). Fourteen variables were normally distributed: three involved the SuL, three the SuH, two the SuP, and the remainder were OL/OH, OP/OH, SuA/OA, SuTipV/SuEndV, SuEndV/OP, and REx. Fourteen variables were non-normally distributed: four inclination angles (α, δ, γ, and β), three the SuL, two shaple indices (ROx and ELx), and the remainder were OP/OL, SuP/OP, SuH/SuP, SuTipV/OP, and OL2/CL.

**Table 2:** Otolith variables that differed significantly among the studied lineages [upper right matrix: Mann-Whitney test, p < 0.05; lower left matrix: ANOVA, p < 0.05, with Tukey HSD (indicated with superscript T) and Dunnett T3 (superscript D) post-hoc tests, depending on homogeneity of variances (Levene’s test, p > 0.05].

	*Gobius* lineage	*Pomatoschistus* lineage	*Acanthogobius* lineage
***Gobius* lineage**		α	α
		δ	δ
		γ	γ
		β	β
		OP/OL	OP/OL
		SuP/OP	SuL/SuH
		SuL/OL	SuH/SuP
		SuL/SuH	OL2/CL
		SuL/OP	ROx
		SuH/SuP	ELx
		SuTipV/OP	
		OL2/CL	
		ROx	
		ELx	
***Pomatoschistus* lineage**	OL/OH^D^		δ
	OP/OH^D^		γ
	SuA/OA^D^		
	SuP/SuTipV^D^		
	SuP/SuEndV^D^		
	SuL/OH^D^		
	SuL/SuTipV^D^		
	SuL/SuEndV^D^		
	SuH/OH^D^		
	SuH/SuTipV^T^		
	SuH/SuEndV^D^		
	SuTipV/SuEndV^T^		
	SuEndV/OP^D^		
	REx^D^		
***Acanthogobius* lineage**	OL/OH^D^	–	
	OP/OH^D^		
	SuA/OA^T^		
	SuP/SuEndV^D^		
	SuL/OH^T^		
	SuL/SuTipV^T^		
	SuL/SuEndV^D^		
	SuH/OH^T^		
	SuH/SuEndV^T^		
	SuTipV/SuEndV^T^		
	SuEndV/OP^D^		
	REx^T^		

These characters indicated clear separation of the *Gobius* lineage from the *Pomatoschistus* (28 variables) and *Acanthogobius* (23 variables) lineages (Table 2, S1 Fig, S1 Table). The *Pomatoschistus* and *Acanthogobius* lineages however, were separated by just two variables (δ and γ). Therefore, δ and γ were the variables that discriminated among all three lineages, whereas 22 variables distinguished the *Gobius* lineage from both the *Pomatoschistus* and *Acanthogobius* lineages.

**Fig 8:**
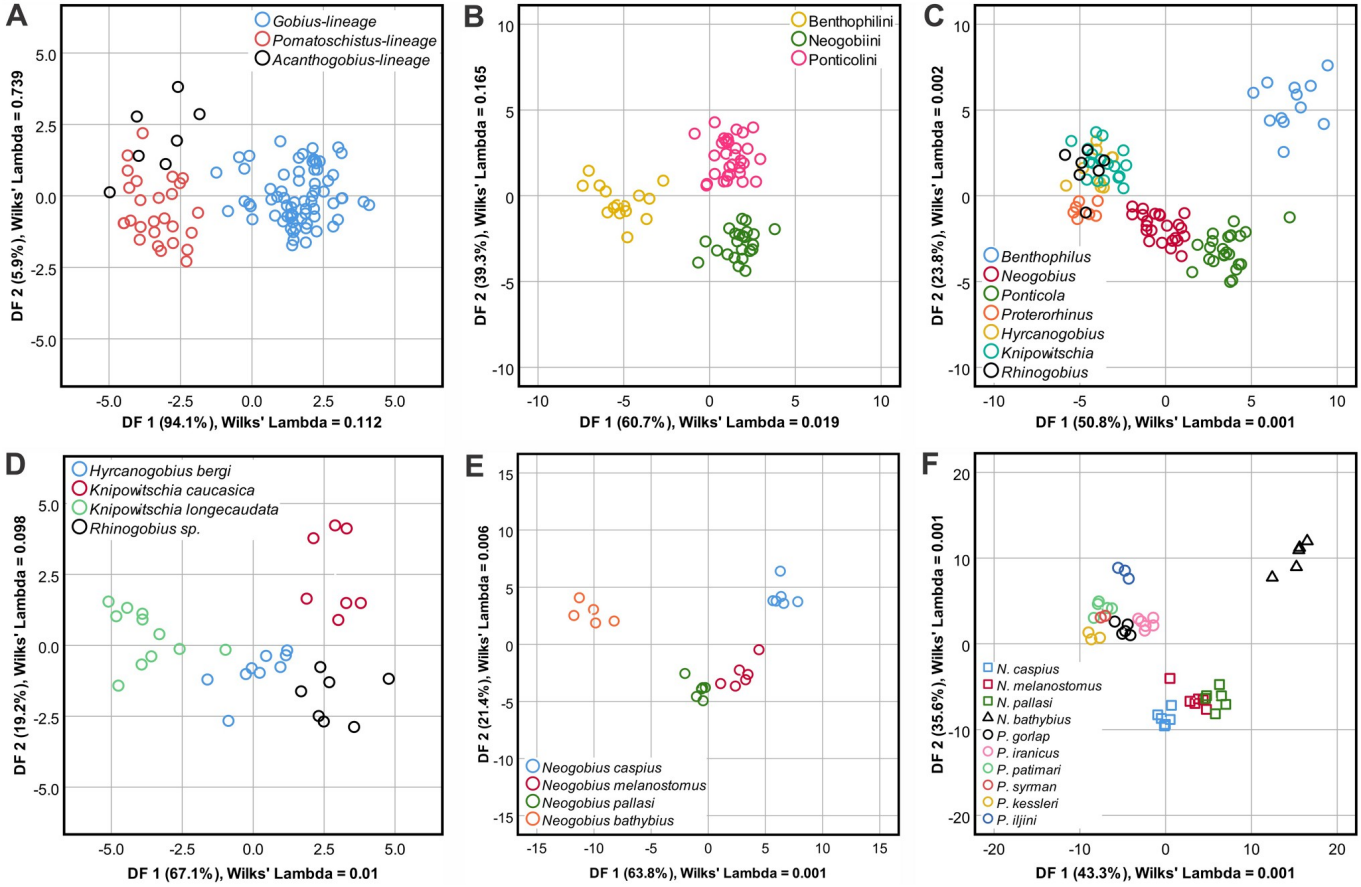
Discriminant function plots based on the otolith variables at taxonomic levels. (a) Lineage, (b) tribe, (c) genus, and (d–f) species in different genera.

The 28 variables were subjected to DFA, yielding two discriminant functions for the variables, DF 1 accounted for 94.1% (eigenvalue: 5.608; λ = 0.112, p < 0.0001) and DF 2 accounted for 5.9% (eigenvalue: 0.353; λ = 0.739, p = 0.13) of the among-group variability. In order of importance, the most significant variables loadings on DF 1 and DF 2 were OL/OH, ROx, OP/OL, OP/OH, SuP/OP, SuP/SuEndV and OP/OH, OL/OH, SuP/SuTipV, SuH/SuTipV, SuH/OH, OP/OL, respectively. The DFA biplot showed clear separation between the *Gobius* lineage and the other two lineages (Fig 8). The proportions of individuals correctly classified into their original lineages were 91.3%, 77.8%, and 57.1% for the *Gobius*, *Pomatoschistus*, and *Acanthogobius* lineages, respectively (Table 3).

**Table 3:** Classification matrix of the canonical discriminant analysis based on otolith variables for the three lineages. Correctly classified samples are shown in bold.

	*Gobius* lineage	*Pomatoschistus* lineage	*Acanthogobius* lineage	Total
***Gobius* lineage**	**91.3**	4.3	4.3	100
***Pomatoschistus* lineage**	0	**77.8**	22.2	100
***Acanthogobius* lineage**	0	42.9	**57.1**	100

Overall classification (cross-validated): 85.4%

#### Variation among the benthophiline tribes

Twenty-nine otolith variables revealed significant differences between at least two of the three benthophiline tribes (Table 4, S2 Fig; S2 Table). Ten variables were normally distributed: three involved the SuL, four the SuH, and the remainder were SuA/OA, SuEndV/OP, and β. Nineteen variables were non-normally distributed: three inclination angles (α, δ, and γ), four involved the SuL, three the SuP, two the OP, two shape indices (ROx and ELx), and the remainder were OL/OH, SuH/SuEndV, SuTipV/OP, SuTipV/SuEndV, and OL2/CL.

**Table 4:** Otolith variables that differed significantly among the three benthophiline tribes [upper right matrix: Mann-Whitney test, p < 0.05; lower left matrix: ANOVA, p < 0.05, with Tukey HSD (indicated with superscript TU) and Dunnett T3 (superscript D3) post-hoc tests, depending on homogeneity of variances (Levene’s test, p > 0.05].

	Benthophilini	Neogobiini	Ponticolini
**Benthophilini**		α	α
		δ	δ
		OL/OH	γ
		OP/OL	SuP/OP
		OP/OH	SuP/SuTipV
		SuP/OP	SuP/SuEndV
		SuP/SuTipV	SuL/OH
		SuP/SuEndV	SuL/SuTipV
		SuL/SuTipV	SuL/SuEndV
		SuL/OP	SuL/OP
		SuH/SuEndV	SuH/SuEndV
		SuTipV/OP	SuTipV/SuEndV
		SuTipV/SuEndV	OL2/CL
		OL2/CL	
		ROx	
		ELx	
**Neogobiini**	SuA/OA^T^		α
	SuL/OL^D^		OL/OH
	SuL/SuH^D^		OP/OL
	SuL/SuP^T^		OP/OH
	SuH/OL^T^		SuP/SuTipV
	SuH/OH^D^		SuP/SuEndV
	SuH/SuTipV^T^		SuL/OH
	SuH/OP^T^		SuL/SuTipV
	SuEndV/OP^T^		SuL/SuEndV
			SuTipV/OP
			ROx
			ELx
**Ponticolini**	SuA/OA^T^	β^D^	
	SuL/OL^D^	SuL/SuH^D^	
	SuL/SuP^T^	SuL/SuP^T^	
	SuH/OL^T^	SuH/OL^T^	
	SuH/OH^T^	SuEndV/OP^T^	
	SuH/SuTipV^T^		
	SuH/OP^T^		
	SuEndV/OP^D^		

Benthophilini was separated from Neogobiini and Ponticolini by 25 and 21 variables, respectively (Table 4, S2 Fig; S2 Table). Neogobiini and Ponticolini were separated by 17 variables. SuL/SuP, SuH/OL, SuEndV/OP, α, SuP/SuTipV, SuP/SuEndV, and SuL/SuTipV discriminated among the three tribes.

The 29 variables were subjected to DFA, which produced two discriminant functions, DF 1 accounted for 60.7% (eigenvalue: 7.836; λ = 0.019, p < 0.0001) and DF 2 for 39.3% (eigenvalue: 5.064; λ = 0.165, p < 0.0001) of the among-group variability. In order of importance, the most significant variables loadings on DF 1 and DF 2 were SuL/OH, SuH/OL, SuH/OH, SuP/SuTipV, SuH/OP, SuH/SuTipV and SuH/OP, SuP/SuTipV, SuH/OL, SuH/OH, SuL/OH, SuL/OH, respectively. The DFA biplot revealed clear separation among the three tribes (Fig 8), for which classification successes were estimated (Table 5). Proportions of individuals that were correctly assigned to their original lineages were 93.3%, 100%, and 90.3% for Benthophilini, Neogobiini, and Ponticolini, respectively.

**Table 5:** Classification matrix of the canonical discriminant analysis based on otolith variables for the three benthophiline tribes. Correctly classified samples are shown in bold.

	Benthophilini	Neogobiini	Ponticolini	Total
**Benthophilini**	**93.3**	0	6.7	100
**Neogobiini**	0	**100.0**	0	100
**Ponticolini**	3.2	6.5	**90.3**	100

Overall classification (cross-validated): 94.2%

#### Variation among the genera

Thirty-one otolith variables revealed significant differences between at least two of the seven genera (Table 6, S3 Fig, S3 Table). Thirteen variables were normally distributed: four variables involved the SuL, four the SuH, and the remainder were γ, OL/OH, OP/OH, SuEndV/OP, and SuH/SuP. Eighteen variables were non-normally distributed: three inclination angles (α, δ, and β), three involved the SuL, three the SuP, two the SuH, two shape indices (ROx and ELx), and the remainder were OP/OL, SuA/OA, SuTipV/OP, SuTipV/SuEndV, and OL2/CL.

**Table 6:** Otolith variables that differed significantly between the studied genera [upper right matrix: Mann-Whitney test, p < 0.05; lower left matrix: ANOVA, p < 0.05, with Tukey HSD (indicated with superscript T) and Dunnett T3 (superscript D) post-hoc tests, depending on homogeneity of variances (Levene’s test, p > 0.05].

	*Benthophilus*	*Neogobius*	*Ponticola*	*Proterorhinus*	*Hyrcanogobius*	*Knipowitschia*	*Rhinogobius*
***Benthophilus***		β	α	δ	α	α	α
		OP/OL	δ	β	δ	δ	δ
		SuA/OA	SuA/OA	OP/OL	β	β	β
		SuP/OP	SuP/OP	SuA/OA	OP/OL	OP/OL	OP/OL
		SuP/SuTipV	SuP/SuTipV	SuP/OP	SuP/OP	SuP/OP	SuP/OP
		SuL/SuTipV	SuP/SuEndV	SuL/OP	SuP/SuEndV	SuP/SuEndV	SuP/SuEndV
		SuL/OP	SuL/SuTipV	SuTipV/OP	SuL/SuEndV	SuL/SuEndV	SuL/SuEndV
		SuH/SuTipV	SuL/SuEndV	OL2/CL	SuTipV/OP	SuTipV/OP	SuTipV/OP
		SuH/SuEndV	SuL/OP	REx	SuTipV/SuEndV	SuTipV/SuEndV	SuTipV/SuEndV
		SuTipV/OP	SuH/SuTipV	ELx	OL2/CL	OL2/CL	OL2/CL
		SuTipV/SuEndV	SuH/SuEndV		REx	REx	REx
		OL2/CL	SuTipV/SuEndV		ELx	ELx	ELx
		ELx	OL2/CL				
		β					
***Neogobius***	OL/OH^D^		α	δ	α	α	δ
	OP/OH^D^		β	OP/OL	δ	δ	β
	SuL/OL^T^		OP/OL	SuP/OP	β	β	SuA/OA
	SuL/SuP^T^		SuP/SuTipV	SuP/SuTipV	OP/OL	SuA/OA	SuP/OP
	SuH/OL^T^		SuP/SuEndV	SuP/SuEndV	SuA/OA	SuP/OP	SuP/SuTipV
	SuH/OH^D^		SuL/SuTipV	SuL/SuTipV	SuP/OP	SuP/SuTipV	SuP/SuEndV
	SuH/OP^T^		SuL/SuEndV	SuL/SuEndV	SuP/SuTipV	SuP/SuEndV	SuL/SuTipV
	SuEndV/OP^T^		SuH/SuTipV	SuL/OP	SuP/SuEndV	SuL/SuTipV	SuL/SuEndV
	ROx^D^		SuH/SuEndV	SuH/SuTipV	SuL/SuTipV	SuL/SuEndV	SuL/OP
			SuTipV/OP	SuH/SuEndV	SuL/SuEndV	SuL/OP	SuH/SuEndV
			ELx	SuTipV/OP	SuL/OP	SuH/SuTipV	SuTipV/SuEndV
				OL2/CL	SuH/SuTipV	SuH/SuEndV	OL2/CL
				REx	SuH/SuEndV	SuTipV/SuEndV	REx
				ELx	SuTipV/SuEndV	OL2/CL	ELx
					OL2/CL	REx	
					REx	ELx	
					ELx		
***Ponticola***	γ^T^	OL/OH^T^		δ	α	α	α
	SuL/OL^D^	OP/OH^T^		β	δ	δ	δ
	SuL/OH^D^	SuL/OH^T^		OP/OL	β	β	β
	SuH/OL^T^	SuL/SuH^D^		SuP/OP	OP/OL	OP/OL	OP/OL
	SuH/OH^D^	SuL/SuP^T^		SuP/SuTipV	SuA/OA	SuA/OA	SuA/OA
	SuH/OP^T^	SuH/OL^T^		SuP/SuEndV	SuP/OP	SuP/OP	SuP/OP
		SuH/SuP^D^		SuL/SuTipV	SuP/SuTipV	SuP/SuTipV	SuP/SuTipV
		SuEndV/OP^T^		SuL/SuEndV	SuP/SuEndV	SuP/SuEndV	SuP/SuEndV
		ROx^T^		SuL/OP	SuL/SuTipV	SuL/SuTipV	SuL/SuTipV
				SuH/SuTipV	SuL/SuEndV	SuL/SuEndV	SuL/SuEndV
				SuH/SuEndV	SuL/OP	SuL/OP	SuL/OP
				SuTipV/OP	SuH/SuTipV	SuH/SuTipV	SuH/SuTipV
				REx	SuH/SuEndV	SuH/SuEndV	SuH/SuEndV
				ELx	SuTipV/OP	SuTipV/SuEndV	SuTipV/SuEndV
					OL2/CL	SuTipV/OP	SuTipV/OP
					REx	OL2/CL	OL2/CL
					ELx	REx	REx
						ELx	ELx
***Proterorhinus***	γ^T^	γ^T^	γ^T^		α	α	α
	OL/OH^D^	OL/OH^T^	OL/OH^T^		δ	δ	β
	OP/OH^D^	OP/OH^T^	OP/OH^T^		β	β	OP/OL
	SuL/OL^T^	SuL/OH^T^	SuL/OH^D^		OP/OL	OP/OL	SuTipV/SuEndV
	SuL/SuH^T^	SuEndV/OP^T^	SuL/SuH^T^		SuA/OA	SuA/OA	SuTipV/OP
	SuL/SuP^T^	ROx^T^	SuL/SuP^T^		SuTipV/SuEndV	SuTipV/SuEndV	OL2/CL
	SuH/OL^T^		SuH/OL^T^		SuTipV/OP	SuTipV/OP	
	SuH/OP^T^		SuH/OH^T^		OL2/CL	OL2/CL	
	SuH/SuP^T^		SuH/SuP^T^				
	SuEndV/OP^T^		SuEndV/OP^T^				
	ROx^T^		ROx^T^				
***Hyrcanogobius***	γ^D^	γ^D^	γ^D^	γ^T^		α	δ
	OL/OH^D^	OL/OH^T^	OL/OH^D^			δ	
	OP/OH^D^	OP/OH^D^	OP/OH^D^			REx	
	SuL/OL^D^	SuL/OL^D^	SuL/OL^T^				
	SuL/SuH^T^	SuL/OH^T^	SuL/OH^D^				
	SuL/SuP^T^	SuH/OH^T^	SuL/SuH^D^				
	SuH/OL^T^	SuEndV/OP^D^	SuL/SuP^T^				
	SuH/OP^T^	ROx^T^	SuH/OH^T^				
	SuH/SuP^T^		SuH/SuP^T^				
	SuEndV/OP^T^		SuEndV/OP^T^				
	ROx^T^		ROx^T^				
***Knipowitschia***	γ^T^	γ^D^	γ^T^	γ^T^	SuL/SuP^T^		δ
	OL/OH^D^	OL/OH^D^	OL/OH^D^	SuH/OL^T^			
	OP/OH^D^	OP/OH^D^	OP/OH^D^				
	SuL/OL^T^	SuL/OL^T^	SuL/OL^T^				
	SuH/OL^T^	SuL/OH^T^	SuL/OH^T^				
	SuH/OP^T^	SuL/SuP^T^	SuL/SuH^T^				
	SuL/OL^D^	SuH/OL^T^	SuH/OH^T^				
	SuL/SuP^T^	SuH/OH^T^	SuH/SuP^T^				
	SuH/OL^T^	SuH/OP^T^	SuEndV/OP^T^				
	SuEndV/OP^T^	SuEndV/OP^D^	ROx^T^				
	ROx^T^	ROx^D^					
***Rhinogobius***	γ^D^	γ^D^	γ^D^	–	γ^D^	γ^D^	
	OL/OH^D^	OL/OH^T^	OL/OH^T^				
	OP/OH^D^	OP/OH^D^	OP/OH^T^				
	SuL/OL^T^	SuL/OL^T^	SuL/OH^T^				
	SuL/SuH^T^	SuL/OH^T^	SuL/SuH^D^				
	SuL/SuP^T^	SuH/OH^T^	SuH/OH^T^				
	SuH/OL^T^	SuEndV/OP^D^	SuH/SuP^D^				
	SuH/OP^T^	ROx^T^	SuEndV/OP^T^				
	SuH/SuP^T^		ROx^T^				
	SuEndV/OP^T^						
	ROx^T^						

*Benthophilus* clearly diverged from the other three benthophiline genera, i.e., *Neogobius* (22 variables), *Ponticola* (19), and *Proterorhinus* (21) (Table 6, S3 Fig, S3 Table). It was distinguished from *Hyrcanogobius*, *Knipowitschia*, and *Rhinogobius*, each by 23 variables. The strongest parameters in this respect were SuL/OL, SuH/OL, SuH/OP, SuP/OP, and OL2/CL, which separated *Benthophilus* from all other genera.

*Neogobius* was clearly distinctive from the other three benthophiline genera, i.e., *Benthophilus* (22 variables), *Ponticola* (20), and *Proterorhinus* (20). It also diverged from *Hyrcanogobius*, *Knipowitschia*, and *Rhinogobius*, by 25, 27, and 22 variables, respectively. The strongest contributors were OL/OH, OP/OH, SuEndV/OP, ROx, SuP/SuTipV, SuL/SuTipV, and SuH/SuEndV in separating *Neogobius* from all other genera.

*Ponticola* differed from the other three benthophiline genera, i.e., *Benthophilus* (19 variables), *Neogobius* (20), and *Proterorhinus* (25). It also diverged from *Hyrcanogobius*, *Knipowitschia*, and *Rhinogobius*, by 28, 28, and 27 variables, respectively. The characters SuL/OH, SuL/SuTipV, SuL/SuEndV, SuH/SuTipV, and SuH/SuEndV best supported the distinction of *Ponticola* from all other genera.

*Proterorhinus* diverged from the other three benthophiline genera, i.e., *Benthophilus* (21 variables), *Neogobius* (20), and *Ponticola* (25). It differed in γ, OL/OH, OP/OH, SuEndV/OP, ROx, δ, OP/OL, SuP/OP, SuL/OP, SuTipV/OP, REx, and ELx. *Proterorhinus* differed from *Hyrcanogobius*, *Knipowitschia*, and *Rhinogobius*, by 9, 10, and 6 variables, respectively. It was distinct from them in α, β, OP/OL, SuTipV/SuEndV, SuTipV/OP, and OL2/CL. The greatest contributing characters were OP/OL and SuTipV/OP, supporting the separation of *Proterorhinus* from all other genera.

*Hyrcanogobius* varied from *Knipowitschia* and *Rhinogobius* by small differences in four (SuL/SuP, α, δ, REx) and two (γ, δ) variables, respectively. *Knipowitschia* and *Rhinogobius* varied in only γ and δ. Therefore, just a single otolith variable (i.e., δ) supported the differentiation of these three genera.

Therefore, of the 31 otolith variables, none distinguished all seven genera. OL/OH, OP/OH, SuEndV/OP, ROx, γ, δ, SuP/OP, REx, and ELx discriminated between two groups, one comprising *Benthophilus* + *Neogobius* + *Ponticola*, and the second group containing *Proterorhinus* + *Hyrcanogobius* + *Knipowitschia* + *Rhinogobius*.

The 31 variables were subjected to DFA; DF 1 accounted for 50.8% (eigenvalue: 17.698; λ = 0.001, p < 0.0001) and DF 2 for 23.8% (eigenvalue: 8.291; λ = 0.002, p < 0.0001) of among-group variability. In order of importance, the most significant variables loadings on DF 1 and DF 2 were SuH/OL, SuH/OP, SuH/OH, SuH/SuEndV, SuP/SuTipV, SuH/SuP and SuL/SuTipV, SuP/SuTipV, SuH/OH, SuH/OP, SuL/OH, OP/OH, respectively. The DFA biplot showed clear separation of *Benthophilus*, *Neogobius*, and *Ponticola*, whereas the other four genera, *Proterorhinus*, *Hyrcanogobius*, *Knipowitschia*, and *Rhinogobius* overlapped (Fig 8). Classification success rates were estimated for the seven genera (Table 7), correctly classifying 100%, 100%, and 100% of *Benthophilus*, *Neogobius*, and *Ponticola*, respectively. For *Proterorhinus*, *Hyrcanogobius*, *Knipowitschia*, and *Rhinogobius*, the proportion of individuals correctly classified into their original genera were 85.7%, 77.8%, 77.8%, and 71.4%, respectively.

**Table 7:** Classification matrix of the canonical discriminant analysis based on otolith variables for the seven genera. Correctly assigned samples are shown in bold.

		1	2	3	4	5	6	7	Total
**1**	*Benthophilus*	**100**	0	0	0	0	0	0	100
**2**	*Neogobius*	0	**100**	0	0	0	0	0	100
**3**	*Ponticola*	0	0	**100**	0	0	0	0	100
**4**	*Proterorhinus*	0	0	0	**85.7**	0	0	14.3	100
**5**	*Hyrcanogobius*	0	0	0	0	**77.8**	22.2	0	100
**6**	*Knipowitschia*	0	0	0	0	11.1	**77.8**	11.1	100
**7**	*Rhinogobius*	0	0	0	0	28.6	0	**71.4**	100

Overall classification (cross-validated): 91%

#### Variation among the *Neogobius* and *Ponticola* species

Twenty-seven otolith variables supported significant differences between at least two of the four *Neogobius* species (Table 8, S4 Fig, S4 Table). Twenty-one variables were normally distributed: five variables involved the SuL, four the SuH, three inclination angles (α, γ, and β), three shape indices (ROx, REx, and ELx), and the remainder were SuTipV/OP, OL/OH, SuA/OA, SuP/OP, OP/OL, and SuTipV/SuEndV. Six variables were non-normally distributed: δ, OP/OH, SuH/SuP, SuEndV/OP, SuP/SuTipV, and SuL/SuTipV.

**Table 8:** Otolith variables that differed significantly between the Caspian *Neogobius* species [upper right matrix: Mann-Whitney test, p < 0.05; lower left matrix: ANOVA, p < 0.05, with Tukey HSD (indicated with superscript T) and Dunnett T3 (superscript D) post-hoc tests, using homogeneity of variances (Levene’s test, p > 0.05].

	*N*. *caspius*	*N*. *melanostomus*	*N*. *pallasi*	*N*. *bathybius*
***N*. *caspius***		δ	OP/OH	δ
		OP/OH	SuEndV/OP	OP/OH
		SuH/SuP		SuEndV/OP
		SuEndV/OP		
***N*. *melanostomus***	SuL/OL^T^		OP/OH	δ
	SuL/SuH^T^		SuP/SuTipV	SuEndV/OP
	SuL/OP^T^		SuH/SuP	
	SuL/SuP^T^			
	SuTipV/OP^T^			
***N*. *pallasi***	γ^T^	SuL/SuP^T^		δ
	OL/OH^D^	SuL/SuH^D^		OP/OH
	SuA/OA^D^	SuH/OL^T^		SuL/SuTipV
	SuP/OP^T^	SuH/OH^T^		
	SuL/OL^T^	SuH/SuTipV^T^		
	SuL/OP^T^	SuH/OP^T^		
	SuH/OL^T^	SuTipV/SuEndV^T^		
	SuH/OP^T^	ROx^T^		
	SuTipV/OP^T^	REx^T^		
	ROx^T^	ELx^T^		
	REx^T^			
	ELx^D^			
***N*. *bathybius***	α^D^	α^D^	α^T^	
	γ^T^	SuA/OA^D^	β^T^	
	OP/OL^T^	SuP/OP^T^	OL/OH^T^	
	SuA/OA^T^	SuL/OH^T^	SuA/OA^D^	
	SuP/OP^T^	SuL/SuH^T^	SuL/OH^T^	
	SuL/OL^T^	SuL/SuP^T^	SuTipV/OP^T^	
	SuL/OH^T^	SuH/OL^T^	SuTipV/SuEndV^T^	
	SuL/OP^T^	SuH/OH^T^	ROx^T^	
	SuH/OL^T^	SuH/OP^T^	REx^D^	
	SuH/OH^T^	SuTipV/OP^T^	ELx^T^	
	SuH/OP^T^			
	SuTipV/OP^T^			

*Neogobius caspius* diverged from *N*. *melanostomus*, *N*. *pallasi* and *N*. *bathybius* by 9, 14 and 15 variables, respectively (Table 8, S4 Fig, S4 Table). *Neogobius melanostomus* differed from *N*. *pallasi* and *N*. *bathybius* by 13 and 12 variables, respectively. *Neogobius pallasi* and *N*. *bathybius* were separated by 13 variables. Of the 27 variables, none separated all four species. SuL/OL, SuL/OP, SuTipV/OP, OP/OH, and SuEndV/OP discriminate *N*. *caspius* from the other three species. SuL/OL, SuL/SuH, and SuL/SuP showed that *N*. *melanostomus* differed from the other three species. The three shape indices ROx, REx, ELx, and OP/OH discriminated *N*. *pallasi* from the other three species. The variables α, δ, SuA/OA, SuL/OH, and SuTipV/OP differed *N*. *bathybius* from the other three species. None of the variables separated all four species.

The 27 variables were subjected to DFA; DF 1 accounted for 63.8% (eigenvalue: 43.557; λ = 0.0001, p < 0.0001) and DF 2 accounted for 21.4% (eigenvalue: 14.617; λ = 0.006, p < 0.001) of among-group variability. In order of importance, the most significant variables loadings on DF 1 and DF 2 were ROx, SuH/OL, SuL/SuH, OL/OH, SuH/OP, SuL/OH and SuH/OP, SuL/SuH, SuH/SuTipV, ROx, SuTipV/OP, OL/OH, respectively. The DFA biplot showed clear separation among three groups (Fig 8), one comprising *N*. *caspius*, the second one *N*. *melanostomus* and *N*. *pallasi*, and the third *N*. *bathybius* alone. The proportion of individuals correctly classified into their original species was 83.3%, 66.7%, 66.7%, and 100% in *N*. *caspius*, *N*. *melanostomus*, *N*. *pallasi*, and *N*. *bathybius*, respectively (Table 9).

**Table 9:** Classification matrix of the canonical discriminant analysis based on otolith variables for the species of *Neogobius* and *Ponticola*. Correctly classified samples are shown in bold.

		1	2	3	4	5	6	7	Total
**1**	*N*. *caspius*	**83.3**	16.7	0	0	0	0	0	100
**2**	*N*. *melanostomus*	16.7	**66.7**	16.7	0	0	0	0	100
**3**	*N*. *pallasi*	0	33.3	**66.7**	0	0	0	0	100
**4**	*N*. *bathybius*	0	0	0	**100**	0	0	0	100
**5**	*P*. *gorlap*	0	0	0	0	**60**	20	20	100
**6**	*P*. *iranicus*	0	0	0	0	16.7	**50**	33.3	100
**7**	*P*. *patimari*	0	0	0	0	20	40	**40**	100

Overall classification (cross-validated): 67.6%

In comparisons among *Ponticola* spp. and *Neogobius bathybius*, 23 otolith variables differed between at least two species (Table 10, S5 Fig, S4 Table). Twenty variables were normally distributed: five variables involved the SuL, two the SuH, two the SuP, two the OP, three shape indices (ROx, REx, and REx), and the remainder were α, γ, OL/OH, SuTipV/OP, SuEndV/OP, and OL2/CL. Three variables were non-normally distributed: δ, SuA/OA, and SuP/SuEndV.

**Table 10:** Otolith variables that differed significantly between the Caspian species of *Ponticola* and *Neogobius bathybius* [upper right matrix: Mann-Whitney test, p < 0.05; lower left matrix: ANOVA, p < 0.05, with Tukey HSD (indicated with superscript T) and Dunnett T3 (superscript D) post-hoc tests, depending on homogeneity of variances (Levene’s test, p > 0.05].

	*N*. *bathybius*	*P*. *gorlap*	*P*. *iranicus*	*P*. *patimari*
***N*. *bathybius***		δ	δ	δ
		SuA/OA	SuA/OA	SuA/OA
		SuP/SuEndV	SuP/SuEndV	SuP/SuEndV
***P*. *gorlap***	α^T^		SuA/OA	–
	OL/OH^D^			
	OP/OH^T^			
	SuP/SuTipV^T^			
	SuL/SuH^T^			
	SuL/SuTipV^T^			
	SuL/SuEndV^T^			
	SuL/SuP^T^			
	SuH/OL^T^			
	SuH/OP^D^			
	SuTipV/OP^T^			
	SuEndV/OP^T^			
	ROx^T^			
	REx^T^			
	Elx^D^			
***P*. *iranicus***	α^T^	γ^T^		–
	OL/OH^T^	SuP/SuTipV^T^		
	OP/OL^T^	SuL/SuTipV^T^		
	OP/OH^D^	REx^T^		
	SuP/SuTipV^D^			
	SuL/OL^T^			
	SuL/SuTipV^D^			
	SuL/SuEndV^D^			
	SuL/SuP^T^			
	SuH/OL^T^			
	SuH/OP^T^			
	SuTipV/OP^T^			
	SuEndV/OP^T^			
	ROx^T^			
	Elx^T^			
***P*. *patimari***	OL/OH^T^	SuP.SuTipV^T^	REx^T^	
	OP/OL^T^	SuL.SuTipV^T^		
	OP/OH^T^	SuTipV/OP^T^		
	SuP/OP^T^	OL2/CL^T^		
	SuL/OL^T^			
	SuL/SuTipV^T^			
	SuL/SuP^T^			
	SuH/OL^T^			
	SuH/OP^T^			
	SuTipV/OP^T^;			
	SuEndV/OP^T^			
	ROx^T^;			
	REx^D^			
	ELx^T^			

The studied species of *Ponticola* diverged from one another by one (*P*. *iranicus* vs. *P*. *patimari*), four (*P*. *gorlap* vs. *P*. *patimari*), or five (*P*. *gorlap* vs. *P*. *iranicus*) variables (Table 10, S5 Fig, S4 Table). *Neogobius bathybius* significantly differed from *P*. *gorlap*, *P*. *iranicus*, and *P*. *patimari* in 17–18 otolith variables. The variables OL/OH, OP/OH, SuL/SuTipV, SuL/SuP, SuH/OL, SuH/OP, SuTipV/OP, SuEndV/OP, ROx, ELx, δ, SuA/OA, and SuP/SuEndV supported separation of *N*. *bathybius* from the three *Ponticola* species. None of the variables distinguished all species.

Species of *Neogobius* and *Ponticola* together were subjected to DFA; DF 1 accounted for 43.3% (eigenvalue: 59.995; λ = 0.0001, p < 0.0001) and DF 2 for 35.6% (eigenvalue: 49.303; λ = 0.0001, p < 0.0001) of among group variability. In order of importance, the most significant variables loadings on DF 1 and DF 2 were SuH/OH, SuTipV/SuEndV, SuTipV/OP, SuH/SuEndV, SuL/OH, OL/OH and SuH/OH, SuP/SuEndV, ROx, SuH/SuEndV, SuH/SuTipV, SuH/OL, respectively. The DFA biplot showed clear separation among three groups (Fig 8), one comprising *Neogobius* spp., the second one *Ponticola* spp., and the third one *N*. *bathybius* alone. Classification success rates were estimated for three *Ponticola* species (*P*. *gorlap*, *P*. *iranicus*, and *P*. *patimari*) (Table 9). The proportion of individuals correctly assigned to their original species was 60%, 50%, and 40%, respectively.

#### Variation in the *Pomatoschistus* and *Acanthogobius* lineages

Twenty-seven otolith variables differed between at least two of the four studied species from the *Pomatoschistus* and *Acanthogobius* lineages (Table 11, S6 Fig, S4 Table). Twenty variables were normally distributed: five variables involved the SuL, four the SuH, two the SuP, three inclination angles (α, γ, and δ), and the remainder were SuTipV/OP, REx, OP/OH, SuA/OA, OL2/CL, and SuEndV/OP. Seven variables were non-normally distributed: two involved the SuL, and the remainder were SuP/OP, SuH/SuTipV, SuTipV/SuEndV, OL/OH, and ELx.

**Table 11:** Otolith variables that differed significantly between the studied species in the *Pomatoschistus* and *Acanthogobius* lineages [upper right matrix: Mann-Whitney test, p < 0.05; lower left matrix: ANOVA, p < 0.05, with Tukey HSD (indicated with superscript T) and Dunnett T3 (superscript D) post-hoc tests, depending on homogeneity of variances (Levene’s test, p > 0.05].

	*H*. *bergi*	*K*. *caucasica*	*K*. *longecaudata*	*Rhinogobius* sp.
***H*. *bergi***		SuP/OP	SuP/OP	–
		SuL/SuEndV	SuL/SuEndV	
		SuH/SuTipV	SuL/OP	
		SuTipV/SuEndV	SuH/SuP	
***K*. *caucasica***	α^T^		OL/OH	SuH/SuTipV
	SuP/SuTipV^T^		SuP/OP	SuTipV/SuEndV
	Sup/SuEndV^T^		SuL/SuEndV	
	SuL/SuP^T^		SuL/OP	
	SuH/OL^T^		SuH/SuTipV	
	SuH/OH^T^		SuTipV/SuEndV	
	SuH/SuEndV^T^		ELx	
	SuH/OP^T^			
	SuTipV/OP^T^			
	REx^T^			
***K*. *longecaudata***	δ^T^	δ^T^		SuL/SuEndV
	SuL/SuH^T^	SuA/OA^T^		
	SuL/SuTipV^T^	SuP/SuTipV^T^		
	SuL/SuP^T^	SuP/SuEndV^T^		
		SuL/OL^T^		
		SuL/OH^T^		
		SuL/SuTipV^T^		
		SuH/OH^T^		
		SuH/SuEndV^T^		
		SuH/OP^T^		
		SuTipV/OP^D^		
		OL2/CL^T^		
***Rhinogobius* sp**.	δ^T^	SuH/OL^T^	δ^D^	
	γ^D^	SuH/OH^T^	SuEndV/OP^T^	
	OP/OH^T^	SuH/SuEndV^T^		
		SuH/OP^T^		
		SuTipV/OP^T^		
		SuEndV/OP^T^		

*Hyrcanogobius bergi* was separated from *Knipowitschia caucasica*, *K*. *longecaudata*, *and Rhinogobius* sp. by 14, 8, and 3 variables, respectively (Table 11, S6 Fig, S4 Table). *Knipowitschia caucasica* differed from *K*. *longecaudata* and *Rhinogobius* sp. by 19 and 8 variables, respectively. *Knipowitschia longecaudata* and *Rhinogobius* sp. were separated by 3 variables. None discriminated *H*. *bergi* from the other three species. The variables SuH/OH, SuH/SuEndV, SuH/OP, SuTipV/OP, SuH/SuTipV, and SuTipV/SuEndV supported *K*. *caucasica* differing from the other three species. δ and SuL/SuEndV discriminated *K*. *longecaudata* from the other three species. None of the variables supported differentiation of *Rhinogobius* sp. from the other three species. Therefore, none of the variables discriminated among the four species.

The 27 variables were subjected to DFA; DF 1 accounted for 67.1% (eigenvalue: 9.017; λ = 0.01, p < 0.0001) and DF 2 for 19.2% (eigenvalue: 2.574; λ = 0.098, p < 0.001) of among-group variability. In order of importance, the most significant variables loadings on DF 1 and DF 2 were SuP/SuEndV, SuH/SuEndV, SuL/SuTipV, SuH/OP, SuH/OH, SuH/SuTipV and SuH/OP, SuH/OL, SuL/SuH, SuH/OH, SuP/SuEndV, respectively. The DFA biplot is shown in Fig 8. The proportion of correct classifications to their original species was 77.8%, 42.9%, 90.9%, and 42.9% in *H*. *bergi*, *K*. *caucasica*, *K*. *longecaudata*, and *Rhinogobius* sp., respectively (Table 12).

**Table 12:** Classification matrix of the canonical discriminant analysis based on otolith variables for the species of the *Pomatoschistus* and *Acanthogobius* lineages. Correctly classified samples are shown in bold.

	*H*. *bergi*	*K*. *caucasica*	*K*. *longecaudata*	*R*. sp.	Total
***Hyrcanogobius bergi***	**77.8**	0	11.1	11.1	100
***Knipowitschia caucasica***	14.3	**42.9**	0	42.9	100
***Knipowitschia longecaudata***	9.1	0	**90.9**	0	100
***Rhinogobius* sp**.	28.6	28.6	0	**42.9**	100

Overall classification (cross-validated): 67.6%

#### Phenotypic relationships based on otolith variables

**Fig 9:**
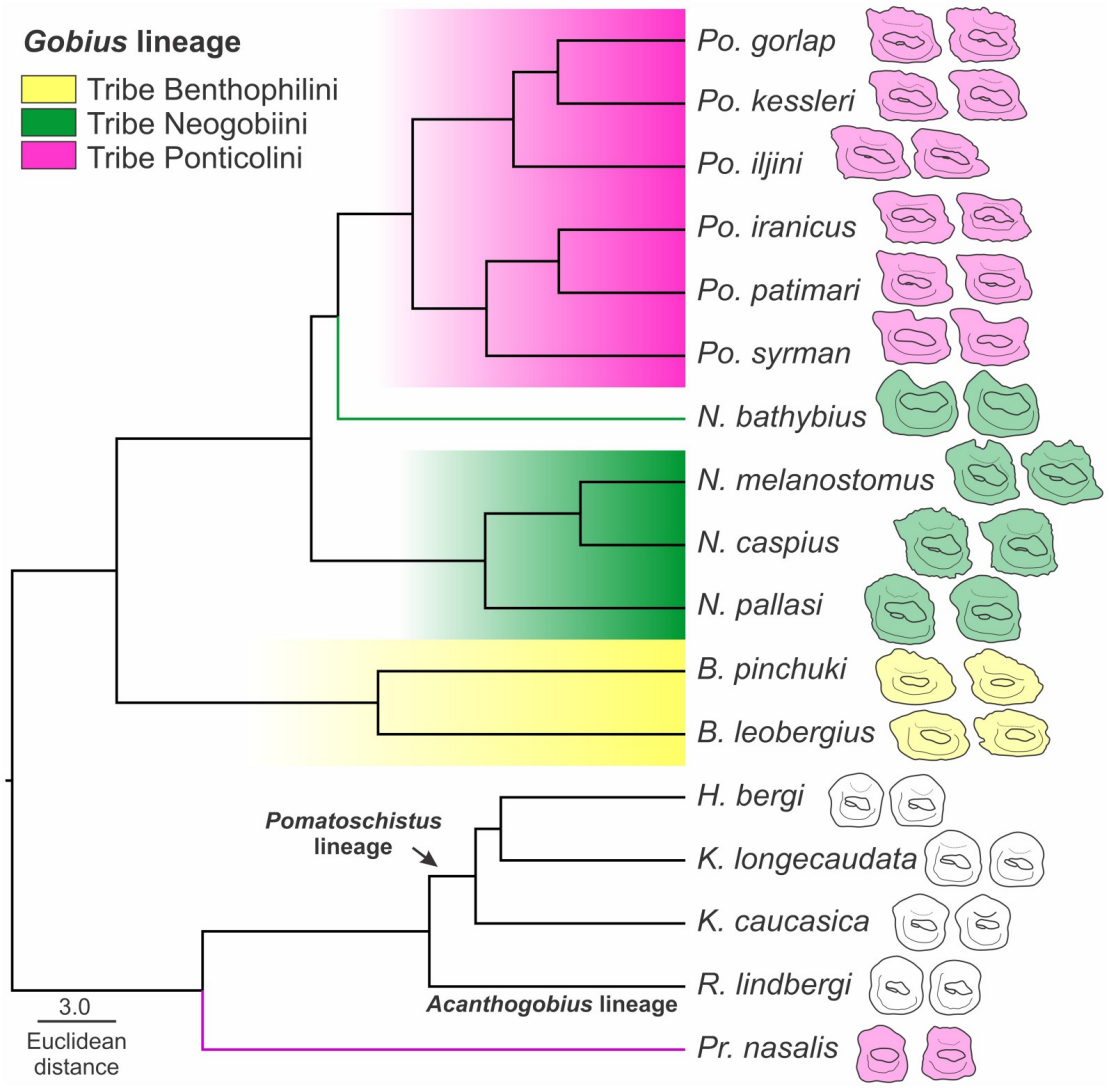
Average linkage dendrogram (using Euclidean distances) showing the phenotypic relationships between otoliths of the studied species, based on all 31 variables. Yellow, Benthophilini; green, Neogobiini; pink, Ponticolini.

A linkage dendrogram based on average Euclidean distances was calculated for 31 otolith variables (Fig 9), which separated the studied fish species into three groups. Group I contained the species in the *Pomatoschistus* and *Acanthogobius* lineages as well as *Proterorhinus nasalis* of the *Gobius* lineage. Group I itself included two sub-groups, with one containing species in the *Pomatoschistus* lineage and *Acanthogobius* lineage, and the second with *Proterorhinus nasalis*. Within the first sub-group, species of the *Pomatoschistus* lineage were monophyletic, sister to *Rhinogobius* sp. The dendrogram shows a closer phenotypic relationship of *Knipowitschia longecaudata* with *Hyrcanogobius bergi*, rather than with *K*. *caucasica*.

Group II included all benthophiline gobies except for *Proterorhinus nasalis*. Four sub-groups were resolved within Group II. The basal sub-group was *Benthophilus leobergius* and *B*. *pinchuki*. Species in the genus *Neogobius* were arranged in two sub-groups: one with *N*. *pallasi*, *N*. *caspius*, and *N*. *melanostomus*, and the other of *N*. *bathybius* alone. The fourth sub-group included all species in the genus *Ponticola* with two sub-clusters: (i) the *Ponticola syrman* group comprising *P*. *syrman*, and two freshwater endemic species, *P*. *iranicus* and *P*. *patimari*, and (ii) the *Ponticola kessleri* group comprising *P*. *iljini*, *P*. *kessleri* and *P*. *gorlap*. *Neogobius bathybius* occupied an intermediate position between the *Neogobius* and *Ponticola* sub-groups.

## Discussion

### Taxonomic significance of otolith morphology in the Caspian gobies

This study aimed to compare the otoliths of extant Caspian gobiids at different taxonomic levels based on detailed descriptions and morphometric method to reveal the taxonomic and phylogenetic information inscribed in their otoliths. The results showed that the otoliths belonging to the *Gobius* lineage are significantly different from those of the *Pomatoschistus* and *Acanthogobius* lineages based on general shape, characteristics of anterior and posterior rims, and otolith variables (overall, 22 otolith variables distinguished the *Gobius* lineage from the other two lineages). Based on morphometric otolith variables, otoliths of the *Pomatoschistus* and *Acanthogobius* lineages from the Caspian Sea basin were largely indistinguishable from each other, separated by small differences in inclination of line connecting preventral angle with tip of posterodorsal projection and inclination angle of posterior rim (see Table 2, S1 Fig, S1 Table). Otolith descriptions however, showed that the otoliths of the two lineages also differed in the shape of their dorsal rim, i.e., being more angular in the *Pomatoschistus* lineage. These results appear to be consistent with the phylogenetic relationships among the three lineages [28]. The overall rate of classification success at this taxonomic level was 85.4%, but 91.3% for the *Gobius* lineage. Within the *Gobius* lineage, the otoliths of the three benthophiline tribes were clearly different based on both methods, with an overall classification success of 94.2%.

The results indicated that the overall shape of the otolith is genus-specific for all Ponto-Caspian gobiid genera, except for *Hyrcanogobius* and *Knipowitschia*, making it the most efficient otolith characteristic for gobiid genus identification in the Caspian Sea basin. The monotypic genus *Hyrcanogobius* and *Knipowitschia* were separated by slight differences in just four variables, i.e., SuL/SuP, α, δ, and REx. *Hyrcanogobius bergi* was originally described by Iljin [50] from the river mouths of the north Caspian Sea as a separate genus *Hyrcanogobius*, because of the reduced condition of the head lateral-line canal system, but later suggested to be congeneric with *Knipowitschia* by Economidis and Miller [51]. However, according to Miller [52], the possession of transverse interorbital papillae rows and a much greater extent of anterior transverse oculoscapular row *tra* (extending downwards to or near the longitudinal suborbital row *b* vs. noticeably short of row *b* in *Knipowitschia*) appear to warrant recognition as a separate genus in any classification based on the head lateral-line system. In addition, paleontological data (otolith) by Bratishko et al. [53] suggest that *Hyrcanogobius* is recognizable as a separate lineage in the fossil record since 11 million years ago. Molecular data of *H*. *bergi* are still missing, however, otolith data support the reassignment of *H*. *bergi* to the genus *Knipowitschia* Iljin, 1927, hereby highlighting the necessity of an integrative molecular, morphological, and paleontological analysis on these species to evaluate their taxonomy.

*Neogobius* presently comprises five valid species: *N*. *caspius*, *N*. *pallasi*, and *N*. *bathybius* are Caspian endemics, *N*. *fluviatilis* in the Black Sea is a sister species of the Caspian *N*. *pallasi*, and *N*. *melanostomus* is native to the Ponto-Caspian basins. The four Caspian species which were well-represented in this study, can be easily distinguished from one other based on otolith morphology results from both descriptive and morphometric methods. The otolith of *N*. *fluviatilis* however, which is poorly represented in this study, is very similar to those of the Caspian *N*. *pallasi*, but a preliminary description of the *N*. *fluviatilis* otolith suggests that they differ with regard to the shape of posterodorsal projection (long and tapering in *N*. *fluviatilis* vs. short and rounded in *N*. *pallasi*) and sulcus (α 14.3° in *N*. *fluviatilis* vs. 16.0–22.5° in *N*. *pallasi*; shallow and ostial lobes weakly developed in *N*. *fluviatilis* vs. relatively deep and ostial lobes well-developed in *N*. *pallasi*). Obviously, more data on the otolith of *N*. *fluviatilis* is needed before drawing any conclusion about its otolith morphology. *Gobius fluviatilis* Pallas, 1814 was originally described in part from near the mouths of rivers falling into the Black Sea and similarly the Caspian Sea. *Neogobius fluviatilis pallasi* (Berg, 1916) was the subspecies described in the Caspian Sea basin. Kottelat and Freyhof [54] recognized *N*. *pallasi* as the Caspian Sea species and restricted *N*. *fluviatilis* to the Black Sea basin. This taxonomic decision was later confirmed by molecular data [55].

*Ponticola* presently comprises eight recognized species in the Caspian Sea basin, six of which were included in this study. The overall shape of otolith in these species is invariably long parallelogram, however, the otoliths of *P*. *syrman* and *P*. *hircaniaensis* are easily distinguishable. The otoliths of *P*. *syrman* and *P*. *hircaniaensis* show concavity in their dorsal rim, however, they are different in their OL/OH (1.40–1.44 vs. 1.22–1.39), OL2/CL (1.65–1.85 vs. 1.59–1.66), SuA/OA (0.09–0.11 vs. 0.13–0.16), SuL/SuH (2.25–3.07 vs. 1.47–1.82), SuH/OL (0.15–0.21 vs. 0.27–0.33), SuH/OH (0.21–0.31 vs. 0.34–0.40), SuH/SuTipV (0.64–0.81 vs. 0.9–1.08), SuH/SuEndV (0.58–0.64 vs. 0.64–0.73), SuTipV/SuEndV (0.79–0.91 vs. 0.65–0.77), ROx (0.65–0.66 vs. 0.69–0.78), and ELx (0.17–0.18 vs. 0.1–0.16) [5]. The otoliths of *P*. *gorlap* differ from those of two south Caspian freshwater endemic species, *P*. *iranicus* and *P*. *patimari* in five (γ, SuP/SuTipV, SuL/SuTipV, REx, and SuA/OA) and four (SuP/SuTipV, SuL/SuTipV, SuTipV/OP, and OL2/CL) otolith variables, respectively. The otoliths of *P*. *gorlap* also differ from those of both species with regard to the incision below the posterodorsal projection (markedly incised vs. slightly incised or not incised), the shape of the predorsal angle (broadly rounded vs. obtuse or orthogonal) and dorsal rim (anteriorly slightly depressed vs. anteriorly not depressed). The otoliths of *P*. *gorlap* are most similar to those of *P*. *iljini*, which may reflect their close phylogenetic relationships and morphological similarities. *Ponticola gorlap* was first identified in the Caspian Sea as *Gobius kessleri* Günther by Kessler [56], who found some morphological differences between the Caspian and typical Black Sea forms. Based on several morphological differences, Iljin [57] suggested that the Caspian gobies from the Mangyshlak region (western Kazakhstan) should be erected as a distinct species, which he described as *Gobius gorlap*. Later, karyological, cranial, head scale, and morphometric data of samples from the Dnieper, Dniester and Volga rivers provided the data supporting specific status of the Mangyshlak samples [58], and subsequently, it was described as a separate species by Vasil’eva and Vasil’ev [59] who regarded the species name *gorlap* as invalid and proposed the new name *iljini*, which later was synonymized with *P*. *gorlap* (Iljin) in a modern phylogenetic systematic study [29]. Vasil’eva et al. [38] reestablished the validity of *P*. *iljini* based on karyological data, but restricted its distribution to the coast of the Mangyshlak Peninsula, western Kazakhstan. As presently understood, *P*. *kessleri*, *P*. *iljini*, and *P*. *gorlap* are closely related and form independent phyletic lineages within a clade of *Ponticola* [29, 38].

The *Ponticola syrman* group comprises two freshwater endemic and cryptic species distinguished from each other mainly based on molecular characters and geographic distributions [7], i.e., *P*. *iranicus* endemic to the upper Sefidroud sub-basin, and *P*. *patimari* endemic to the western freshwater habitats of the south Caspian sub-basin. PCA and DFA plots for the meristic and morphometric data also showed a clear separation of the two species [7]. Our otolith morphometric variables, inclination angles, and classical shape descriptors show that the otoliths of *P*. *iranicus* and *P*. *patimari* are only slightly different in their REx. Also, the otolith shape analysis of 213 specimens representing six sub-basin samples of these species presented a high level of shape variation which did not show congruence with their taxonomy and phylogeographic structure [7]. Studies suggest that while genetics constrain the overall shape of the otolith itself, environmental conditions may eventually alter the rates of somatic and otolith growth, which in turn may affect otolith shape.

Tadpole-gobies of the genus *Benthophilus* are a group of 21 poorly known species from the fresh and brackish waters of the Caspian and Black Sea basins, including the Sea of Azov [37]. Boldyrev and Bogutskaya [41] recognized 20 species and assigned them to four phenotypic groups (i.e., I, II, III, and IV), based on differences in size, arrangement and counts of dermal ossifications, fin ray counts, and body shape. However, the phylogenetic integrity of these groups has never been tested, since the phylogeny of the genus is poorly known and genetic data are available for only a few species [29, 30]. The phylogenetic integrity of the four phenotypic groups established by Boldyrev & Bogutskaya [41] was questioned by Kovačić et al. [37], since a newly described species *B*. *persicus* Kovačić, Esmaeili, Zarei, Abbasi & Schliewen, 2021 featured a mix of characters of phenotypic groups II and III. Interestingly though, the phylogenetic inferences of Neilson and Stepien [29] and Zarei et al. [30] estimated a closer relationship between a group II member (a specimen identified as *B*. *abdurahmanovi*) and the group I member (a specimen identified as *B*. *granulosus* Kessler, 1877), than to three other group II species used in their analyses (identified as *B*. *mahmudbejovi* Ragimov, 1976, *B*. *stellatus* and *B*. *leobergius*). Our otolith data also question the phylogenetic integrity of the four phenotypic groups defined by Boldyrev & Bogutskaya [41]: the otoliths of the species examined here comprised four phenotypic groups based on their overall shapes, which did not show congruence to their hypothesis: group 1: *B*. *leobergius* (II); group 2: *B*. *pinchuki* (III), *B*. *microcephalus* (II), *B*. *stellatus* (II), *B*. *baeri* (IV); group 3: *B*. *durreli* (II); and group 4: *B*. *abdurahmanovi* (II). However, we consider our results preliminary and await substantially increased taxon sampling and a thorough phylogenetic analysis of the species.

### Otolith data suggest the monophyly of neogobiin gobies

**Fig 10:**
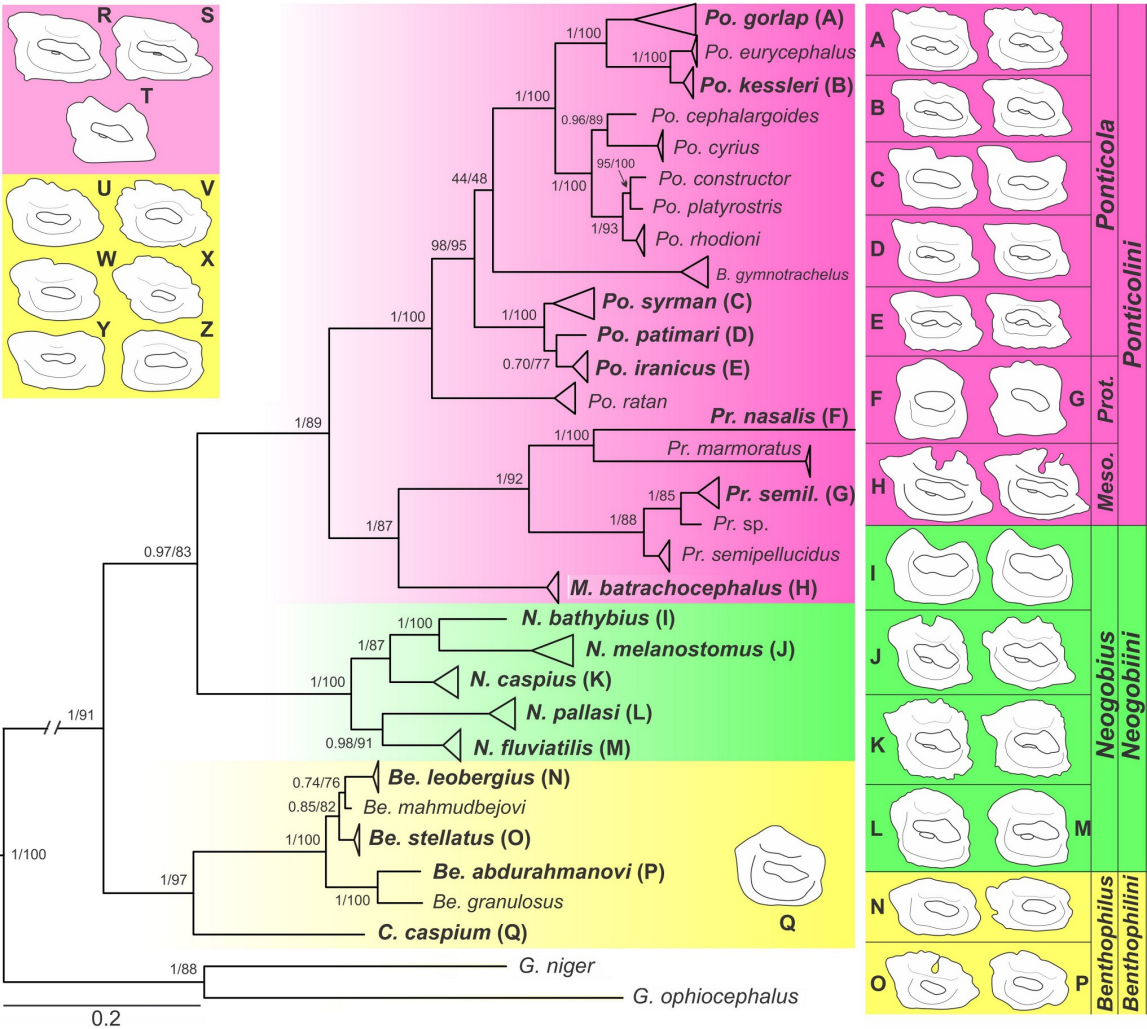
Phylogenetic mapping of general otolith morphology for the studied benthophiline species (COI phylogeny modified from Zarei et al. [30]). Each tribe is represented by a different color: yellow, Benthophilini; green, Neogobiini; pink, Ponticolini. BI posterior probability/ML bootstrap support values are indicated beside the nodes. Double bar at the root indicates that branch has been reduced in length and is not proportional to the scale. Alphabetical letters inside parentheses after the species names (A–Q: 17 species) refer to their otolith drawings on the right. Otolith drawings on the upper left corner belong to *Ponticola iljini* (R–S), *Mesogobius nonultimus* (T), *Benthophilus pinchuki* (U), *B*. *macrocephalus* (V), *B*. *durrelli* (W), *B*. *baeri* (X), *Anatirostrum profundorum* (Y), and *Benthophiloides brauneri* (Z), which have not been phylogenetically analysed.

Molecular phylogenies have supported a monophyletic clade comprising the neogobiin and benthophilin gobies [29, 60]. This monophyletic clade contains three distinctive sub-clades designated as tribes Benthophilini, Neogobiini, and Ponticolini. The phylogenetic placement of Benthophilini has been inconsistent among analyses. The two combined mito-nuclear analyses resolve Benthophilini as the sister clade to Ponticolini [29, 60], however, this relationship had mixed support from different analysis methods, represented a short internal branch, having low internode certainty and gene support frequency. In the *cyt b* analysis of Neilson and Stepien [29], Benthophilini was the sister clade to Neogobiini, yet in their COI analysis and also the *cyt b* analysis of Medvedev et al. [61], Benthophilini again grouped with Ponticolini. The COI analysis of Zarei et al. [30] resolved Benthophilini as the sister clade to Ponticolini + Neogobiini (Fig 10). The latter phylogenetic hypothesis was also supported by our otolith data: Benthophilini differed significantly from Neogobiini and Ponticolini by 25 and 21 variables, respectively, whereas Neogobiini and Ponticolini were separated by 17 variables. The average linkage dendrogram based on the Euclidean distance for the otolith variables also clustered Benthophilini as the sister clade of Neogobiini + *Ponticola*. This phylogenetic hypothesis also agrees with Schwarzhans et al. [12] based on articulated skeletons and otoliths of fossil gobiids: (i) both the neogobiin and benthophilin subgroups were represented by ‘‘primitive” extinct genera (i.e., †*Proneogobius* and †*Protobenthophilus*) considered to be the sister group to all extant members of their respective subgroups, and (ii) the origin and separation of the two subgroups likely links to the segregation of the Eastern Paratethys during the Langhian stage (16–13.6 Ma) of the middle Miocene. Nevertheless, additional genomic, morphological, and taxonomic sampling are needed to further resolve relationships among the major Ponto-Caspian endemic gobiid lineages.

### Systematics of *Neogobius bathybius*

The taxonomic status of *Neogobius bathybius* has been controversial. *Gobius bathybius* Kessler (1877) originally was described from the Svinoi Island, Caspian Sea. The name *Chasar* appeared in print for the first time in Berg [62] as a subgenus of *Neogobius* to accommodate *bathybius*. Berg [62] provided a brief description of the species as *Neogobius* (*Chasar*) *bathybius*, but did not define the genus-group category. Although Vasil’eva [63] stated that Iljin [64, 65] used this subgeneric name for *bathybius*, a search of the latter publications by Miller [66] found this species mentioned only as being *incertae sedis*, but without reference to any previous use of the name *Chasar* or to a definition by Iljin. Both Iljin [57] and Ragimov [67] used the name at a subgeneric level, but again provided no diagnosis. Pinchuk and Ragimov [68], in their redescription of *bathybius*, placed this species in *Neogobius* without a subgenus. The first subgeneric diagnosis of *Chasar*, with indication of the type and only species, thus appears to be by Vasil’eva [63]. Detailed osteological comparison with other gobiid taxa by Vasil’eva [63] suggested that *bathybius* occupied a distinct subgeneric position. The monotypic genus *Chasar* was recognized as a valid taxon by Miller [66], on the basis of the head sensory papillae patterns noted by Pinchuk and Ragimov [68] and Vasil’eva [63]. The resulting paraphyly of *Neogobius sensu lato* [62] however was changed in Neilson and Stepien’s [29] revised classification, by elevating two of Iljin’s [64] subgenera to genus rank, i.e., *Babka* and *Ponticola* for the remainder of the ‘neogobiin’ species. Neilson and Stepien [29] included *bathybius* in *Ponticola* in their study without further justification, since they lacked *bathybius* specimens to sequence and did not hypothesize a nominal genus.

**Fig 11:**
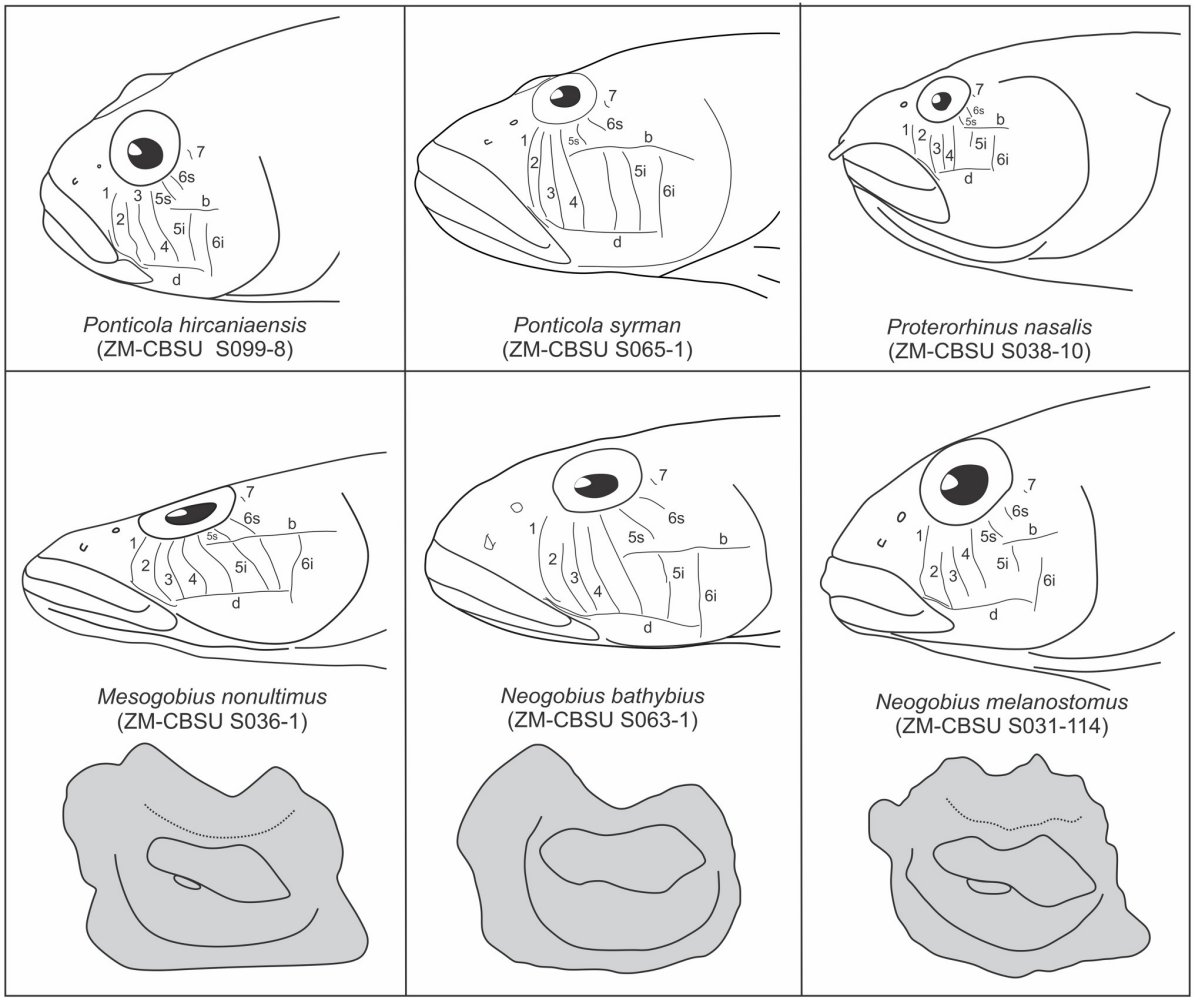
Head lateral line system (left side, suborbital rows) of *Ponticola*, *Proterorhinus*, *Mesogobius*, and *Neogobius*. For comparison, the head lateral line system and otolith drawings of *M*. *nonultimus*, *N*. *bathybius*, and *N*. *melanostomus* are shown in the bottom row.

Recent phylogenetic analyses by Zarei et al. [30] and Tajbakhsh et al. [69] provided support for the reclassification of *Gobius bathybius* from *Ponticola* to *Neogobius sensu stricto* [29]. *Neogobius bathybius* however, differs from the other four *Neogobius* species in its cheek sensory papillae pattern by possessing one additional transverse row before row *b* (i.e., five transverse suborbital rows before row *b*; Fig 11) [68]. The presence of eight transverse suborbital rows and five before row *b* might be interpreted as a synapomorphy with *Mesogobius*, but *N*. *bathybius* possesses two transverse rows below row *b*, a plesiomorphic feature shared with *Neogobius*, *Ponticola* (except for *P*. *syrman* that has three rows), and *Proterorhinus*; this differs from *Mesogobius*, which has three rows. The suborbital lateral line system pattern of *Mesogobius* and *N*. *bathybius* do not completely match and might be attributed to parallel evolution or to two step development, where the first step was the synapomorphy of an additional transverse suborbital row in front of suborbital row *b*, and the second step was the addition of one more transverse suborbital row below row *b*. In terms of otolith morphology, the otoliths of all *Neogobius* species except for *bathybius* are characterized by having a square-rhomboid (*N*. *caspius* and *N*. *melanostomus*) to a discoid-rhomboid (*N*. *fluviatilis* and *N*. *pallasi*) shape, a convex and regularly curved dorsal rim, a posterodorsal projection that is usually long and narrow, tapering or rounded, and strongly bent outwards, δ 24.3–33.4°, α 14.7–22.5°, and presence of subcaudal iugum and dorsal depression. On the other hand, the *N*. *bathybius* otolith is characterized by having a two-humped long rectangle shape, a highly positioned posterior hump, a dorsal rim with a deep broad V-shaped concavity, a posterodorsal projection that is bulky and very broad and does not bend outwards, δ 13.5–22.1°, α 2.1–10.6°, and absence of subcaudal iugum and dorsal depression. Among the otoliths of all Ponto-Caspian endemic gobiids studied here, a dorsal rim with two angular humps (with the posterior hump being highly positioned) and a deep broad V-shaped concavity, and a bulky and very broad posterodorsal projection are found in only one other species, *Mesogobius nunultimus*. Zarei et al. [30] suggest that as an alternative to the mitochondrial phylogenetic hypothesis [30, 69], an ancient hybridization scenario between *Neogobius melanostomus* and *Mesogobius nunultimus* might have led to the same phylogenetic pattern as the hypothesized sister group relationship between *bathybius* and *N*. *melanostomus*. In addition to the suborbital papillae pattern and the otolith shape, *N*. *bathybius* shows several other intermediate morphological and ecological characteristics (Table 13). However, its intermediate characters (best exemplified by the intermediate number of vertebrae, second dorsal and anal fin elements, longitudinal scale rows, head, body and caudal peduncle depths, longevity, and migration) and those completely different from the characters of the putative parental species (e.g., first dorsal elements, growth, habitat, depth, and spawning period; Table 13) could be the result of hybridization and later isolating evolution, as well as the outcome of other scenarios. Therefore, we suggest a chromosome analysis and genomic approach to resolve the generic classification of Neogobini and Ponticolini.

**Table 13:** Some morphological and ecological characteristics of *Neogobius melanostomus*, *N*. *bathybius*, and *Mesogobius nonultimus* [70–7,2].

	*N*. *melanostomus*	*N*. *bathybius*	*M*. *nonultimus*
**First dorsal fin spines**	6	7	6
**Second dorsal fin branched rays**	12–17	14–16	16–19
**Anal fin branched rays**	10–14	11–13	15–18
**Longitudinal scale rows**	45–57	55–65	76–83
**Vertebral column** *	32–33	35	36
**Head depth at nape**	18.8–24.2% SL	15.2–20.1% SL	11.9–16.2% SL
**Maximum body depth**	20.7–26.7% SL	16.3–23.6% SL	13.8–19.3% SL
**Least depth of caudal peduncle**	10.4–13.5% SL	6.9–9.0% SL	5.7–7.3% SL
**Growth**	up to 124 mm TL	up to 290 mm TL	up to 174 mm TL
**Habitat**	euryhaline (0–40.5 ‰)	pleiomesohaline (10–16.5 ‰)	mesohaline (3–16.5 ‰)
**Depth**	usually found in coastal shallows (1.5–20 m)	deep waters (75 m to deeper)	relatively deep waters (24–54 m)
**Migration**	during the breeding season, there is a migration (initiated by the males) close inshore to 0.2–0.5 m, but in winter the species may move down usually to 20–30 m or even to 60–70 m	sexually mature males begin approaching the coast in the first half of March or April; females with ripe eggs start to enter shallower water in later April, with greater numbers in May-June; in contrast to other species, it migrates immediately after spawning to deeper areas of more than 10–15 m	feeding, overwintering, and breeding in relatively deep waters, without a spring onshore migration
**Spawning period**	April to September	mid-June until July	late March until mid-April
**Longevity**	3 (male) to 5 (female) years	4 years	no data, but 7–8 years in *M*. *batrachocephalus* [34]

*Unpublished data.

### Phylogenetic placement of *Anatirostrum* and *Benthophiloides*

The morphologically well-defined benthophilin group is characterized by several apomorphies: (i) suborbital row *5i* longer than row *6i*, extending below rear termination of row *d*, with the row *6i* separated from row *d*, (ii) suborbital row *4* not ascending above the level of row *b*, (iii) interorbital papillae present, (iv) loss of all head canals, (v) a tubular anterior nostril without a rim process, and (vii) absence or, at least, reduction in scale cover, with no squamation on head. Hitherto, the two phylogenetically studied benthophilin genera, *Benthophilus* and *Caspiosoma*, formed a monophyletic clade referred to by Neilson and Stepien [29] as Benthophilini. The otoliths of the benthophilin group are characterized by a reduced, shallow sulcus that lacks well-developed ostial lobes and has no subcaudal iugum. Each of the four benthophilin genera possess a distinct otolith shape, reflecting their generic classification and phylogenetic affinities: long elliptical in *Benthophilus*, right trapezoid in *Anatirostrum*, long rectangle in *Benthophiloides*, and pentagonal in *Caspiosoma*.

*Caspiosoma caspium*, having the most distinctive otolith shape among the four benthophilin genera, has a basal phylogenetic placement within Benthophilini [29]. Absence of scales or scale-like derivatives in *Caspiosoma* is a reductive feature shared with *Benthophiloides*; however, the otoliths of the two genera differ chiefly in the shape of dorsal rim above the cauda (angular vs. convex and gently curved, highest above cauda) and the inclination of ostium (inclined at 7.5° vs. not inclined). Also, a significant number of morphological features link *Benthophilus* with *Anatirostrum* [34], but *Anatirostrum* lacks the chin barbel and dermal fold at the jaw angle of *Benthophilus* as well as the large tubercles seen in *Benthophilus*. Within the benthophilin group, *Anatirostrum* shows a number of autapomorphies including an additional suborbital row below level of suborbital longitudinal row *b*, an elongated and duck-bill shaped snout with posterior nostrils displaced well anterior to the orbit, and a complete sequence of pterygiophores between the first and second dorsal fins. Furthermore, the otoliths of *Anatirostrum* and *Benthophilus* differ mainly in the dorsal rim’s curvature (horizontal to slightly concave vs. convex and gently curving), and the shape of the predorsal angle (orthogonal and slightly raised vs. obtuse and markedly depressed). Molecular data of *Benthophiloides* and *Anatirostrum* are still missing, however, these mosaic patterns of morphological features indicate relatively divergent but sister group relationships between *Caspiosoma* and *Benthophibides*, and between *Benthophilus* and *Anatirostrum*.

## Conclusion

The results indicated high taxonomic efficiency of otolith morphology combined with morphometry at different taxonomic levels for the Ponto-Caspian gobiids. Separation of otoliths at different taxonomic levels requires consideration of different morphological characters and otolith variables [46]. This was also the case in our study. In addition, it also appears that these qualitative and quantitative otolith data contain important phylogenetic signals, however, more studies are needed to complete these evaluations and confirm our otolith study findings.

## Supporting information

S1 Checklist Ethical, cultural, and scientific considerations specific to inclusivity in global research. (DOCX)

S1 Fig Box plots of 28 otolith variables that were useful in the separation of the three lineages. *G*.*L*., *Gobius* lineage; *P*.*L*., *Pomatoschistus* lineage; *A*.*L*., *Acanthogobius* lineage.
(TIF)

S2 Fig Box plots of 29 otolith variables that were useful in the separation of the three benthopheline tribes. Green, normally distributed variables; blue, non-normally distributed variables.
(TIF)

S3 Fig Box plots of 31 otolith variables that were useful in the separation of the seven genera. *Be*., *Benthophilus*; *Ne*., *Neogobius*; *Po*., *Ponticola*; *Pr*., *Proterorhinus*; *Hy*., *Hyrcanogobius*; *Kn*., *Knipowitschia*; *Rh*., *Rhinogobius*. Green, normally distributed variables; blue, non-normally distributed variables.
(TIF)

S4 Fig Box plots of 27 otolith variables that were useful in the separation of the studied species in the genus *Negobius*. *N*. *c*., *Neogobius caspius*; *N*. *m*., *Neogobius melanostomus*; *N*. *p*., *Neogobius pallasi*; *N*. *b*., *Neogobius bathybius*. Green, normally distributed variables; blue, non-normally distributed variables.
(TIF)

S5 Fig Box plots of 23 otolith variables that were useful in the separation of the studied species of *Ponticola* and *Negobius bathybius* (*N*. *b*.). *P*. *g*., *Ponticola gorlap*; *P*. *i*., *Ponticola iranicus*; *P*. *p*., *Ponticola patimari*. Green, normally distributed variables; blue, non-normally distributed variables.
(TIF)

S6 Fig Box plots of 27 otolith variables that were useful in the separation of the studied species in the *Pomatoschistus* and *Acanthogobius* lineages. *H*. *b*., *Hyrcanogobius bergi*; *K*. *c*., *Knipowitschia caucasica*; *K*. *l*., *Knipowitschia longecaudata*; *R*. sp., *Rhinogobius* sp. Green, normally distributed variables; blue, non-normally distributed variables.
(TIF)

S1 Table Calculated otolith variables for the three gobiid lineages. (XLSX)

S2 Table Calculated otolith variables for the three benthopheline tribes. (XLSX)

S3 Table Calculated otolith variables for the seven gobiid genera. (XLSX)

S4 Table Calculated otolith variables for the studied species. (XLSX)
