# Supplementary figures and images for: Otoliths of Caspian gobies (Teleostei: Gobiidae): Morphological diversity and phylogenetic implications
Source: PLoS One. 2023 May 15;18(5):e0285857. doi: 10.1371/journal.pone.0285857 (PMC10184949; doi:10.1371/journal.pone.0285857)

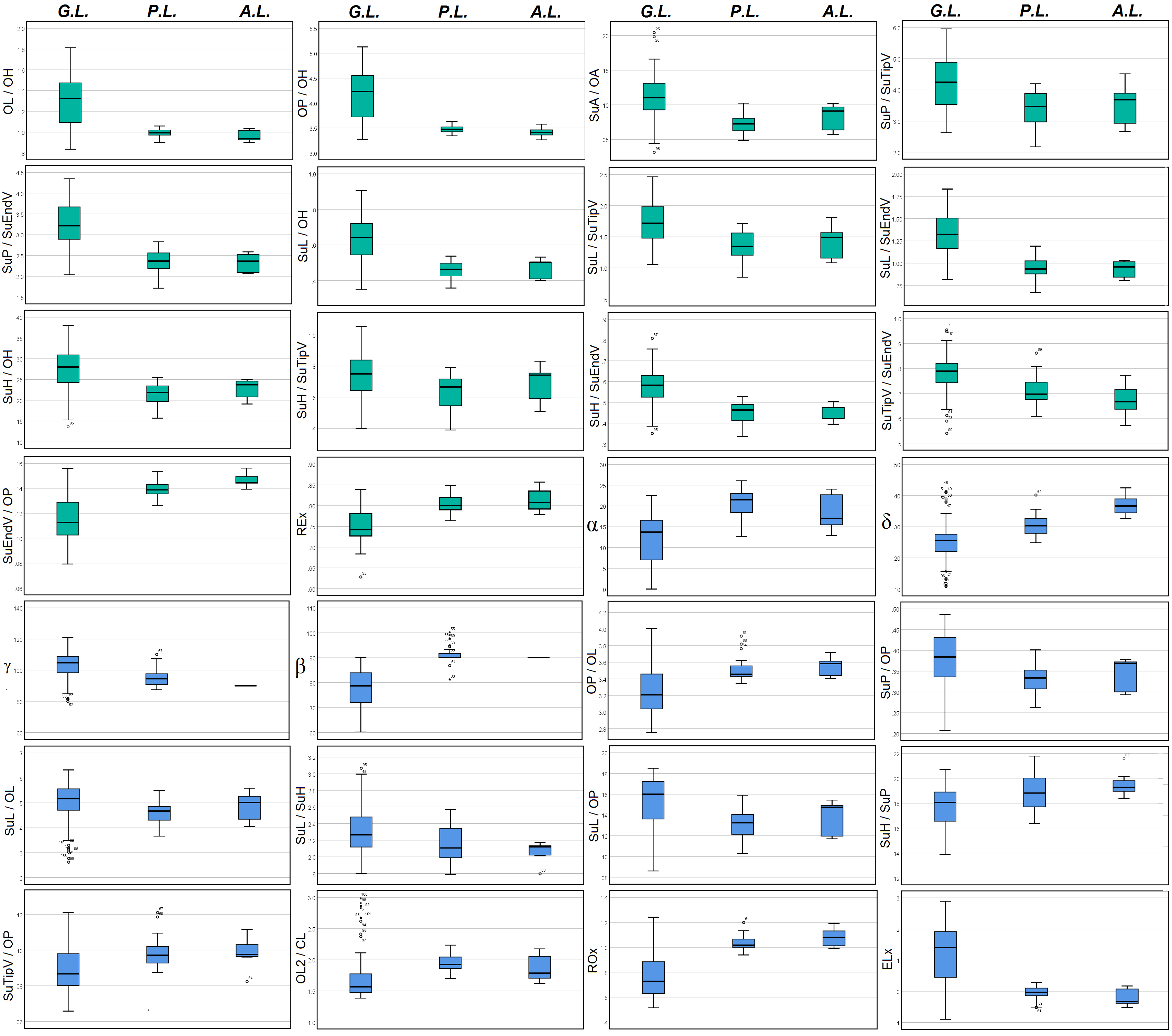

Supplement: S1 Fig — G.L., Gobius lineage; P.L., Pomatoschistus lineage; A.L., Acanthogobius lineage. (TIF) [file pone.0285857.s002.tif]

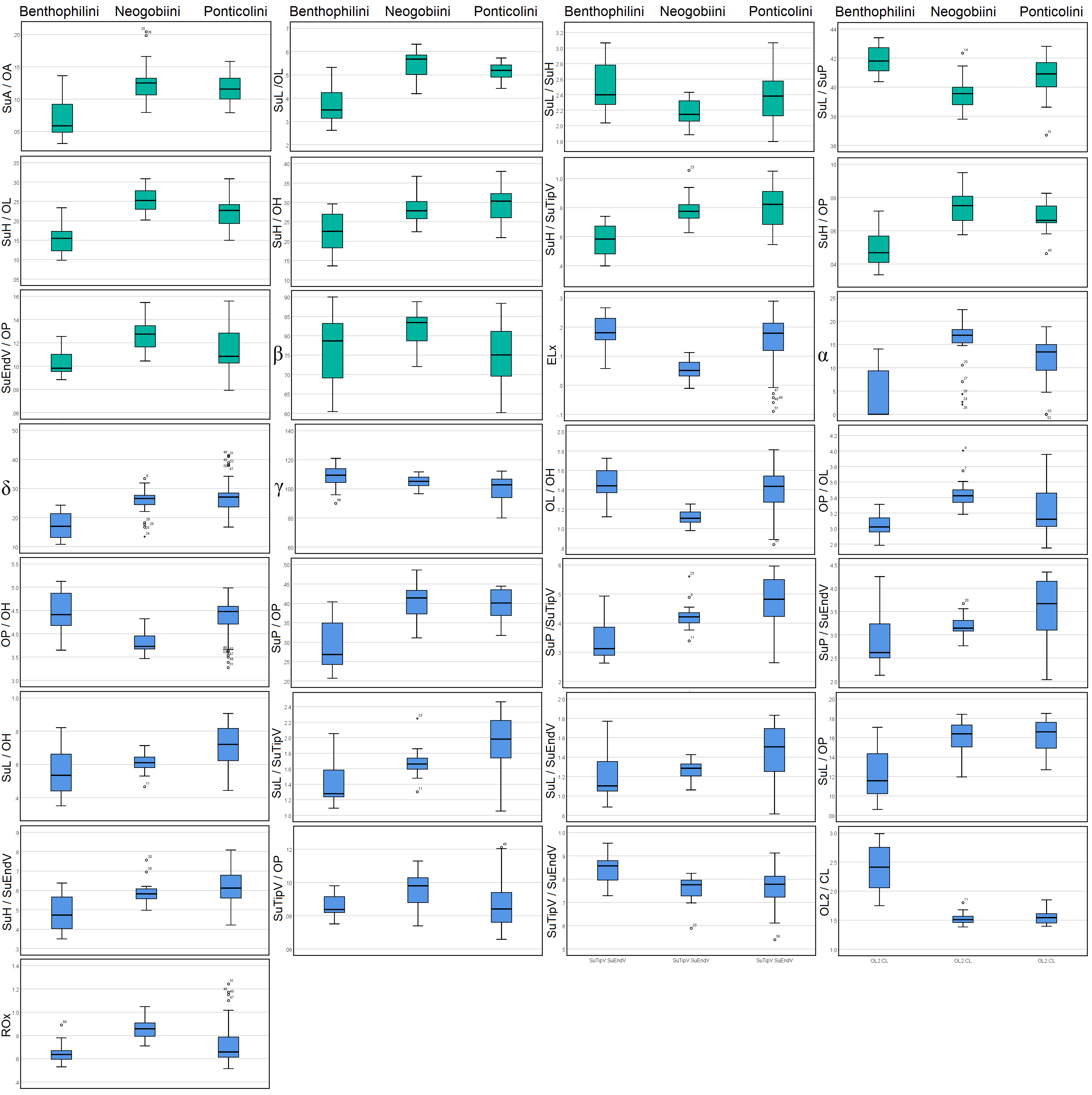

Supplement: S2 Fig — Green, normally distributed variables; blue, non-normally distributed variables. (TIF) [file pone.0285857.s003.tif]

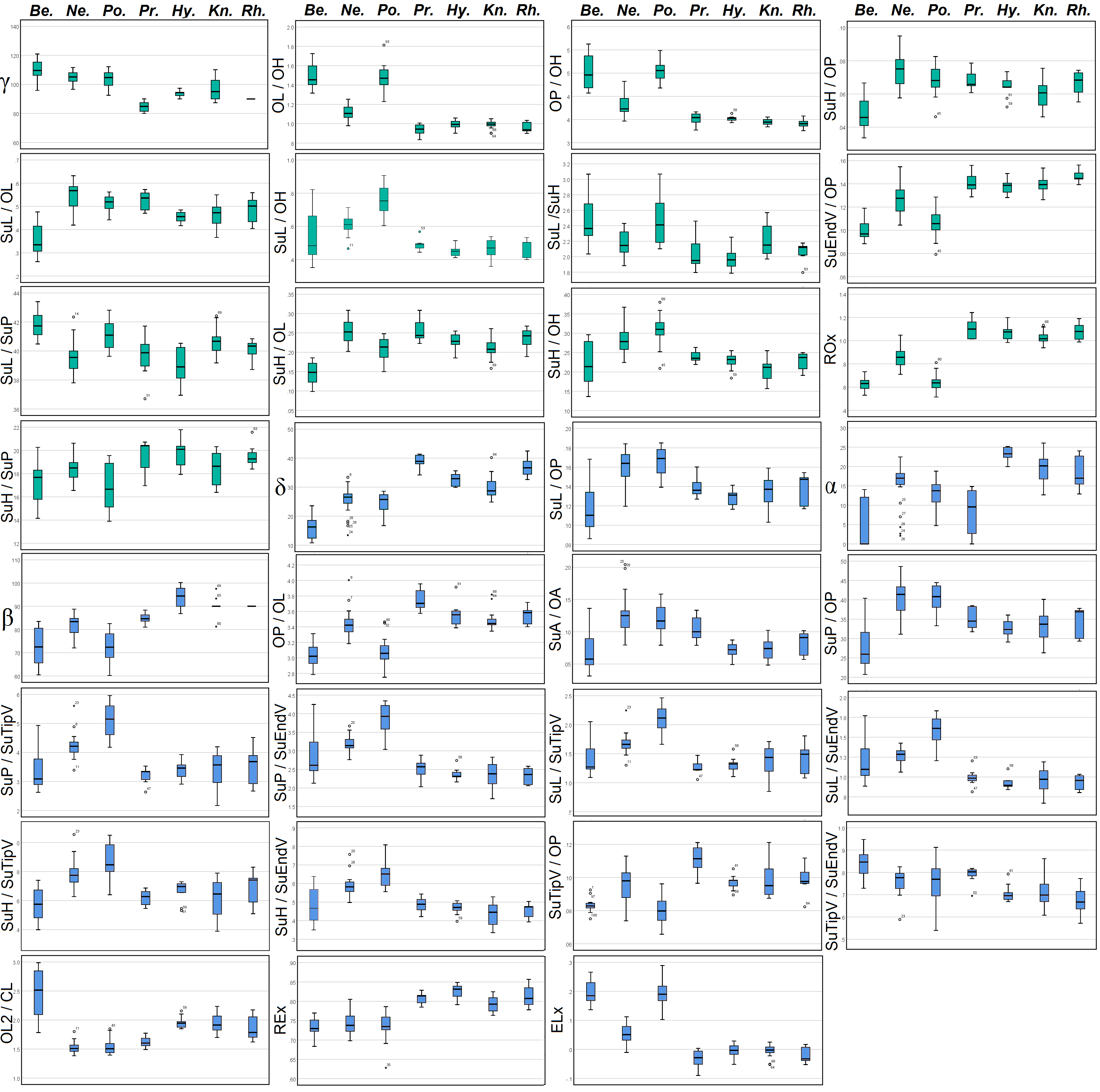

Supplement: S3 Fig — Be., Benthophilus; Ne., Neogobius; Po., Ponticola; Pr., Proterorhinus; Hy., Hyrcanogobius; Kn., Knipowitschia; Rh., Rhinogobius. Green, normally distributed variables; blue, non-normally distributed variables. (TIF) [file pone.0285857.s004.tif]

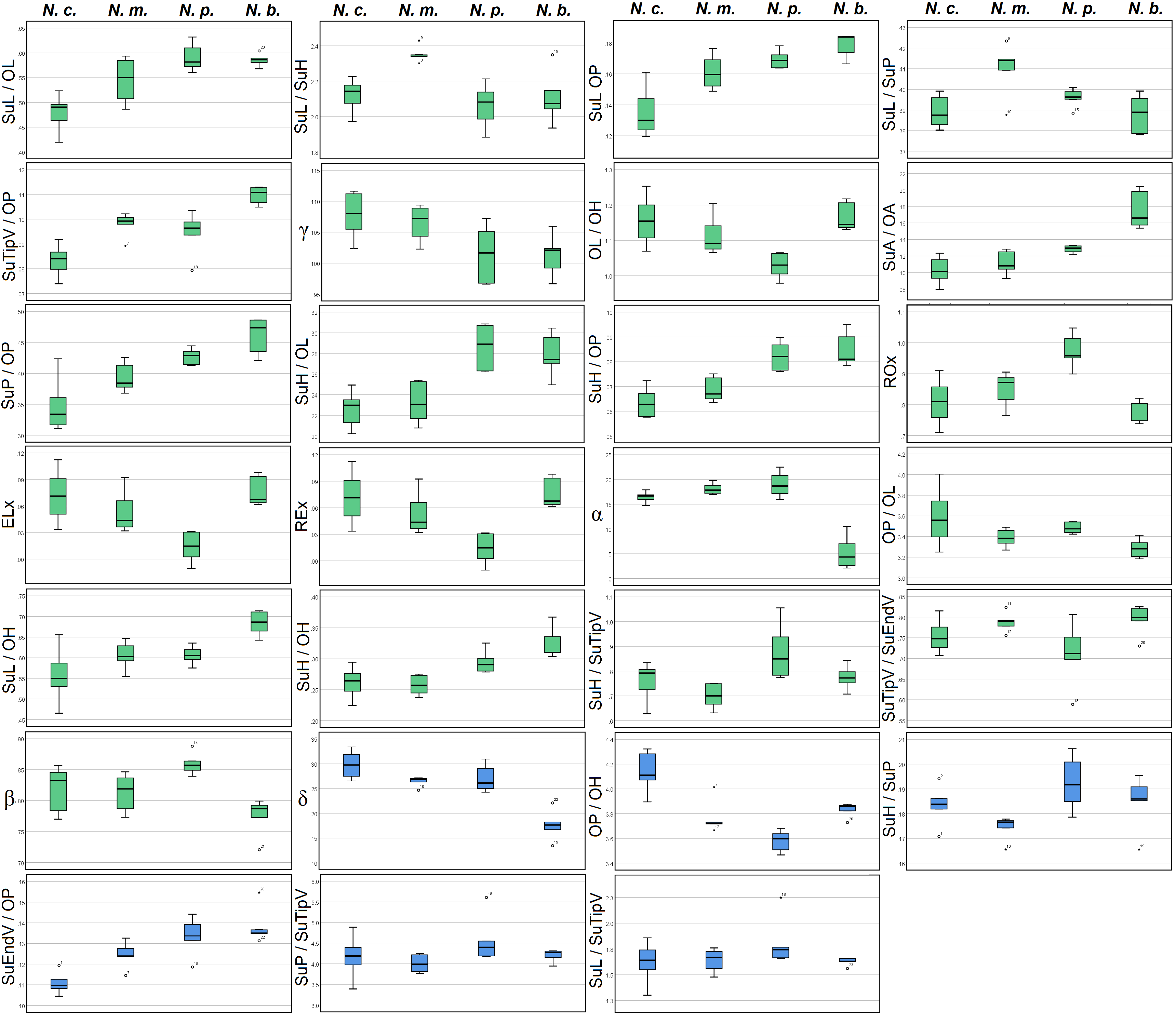

Supplement: S4 Fig — N. c., Neogobius caspius; N. m., Neogobius melanostomus; N. p., Neogobius pallasi; N. b., Neogobius bathybius. Green, normally distributed variables; blue, non-normally distributed variables. (TIF) [file pone.0285857.s005.tif]

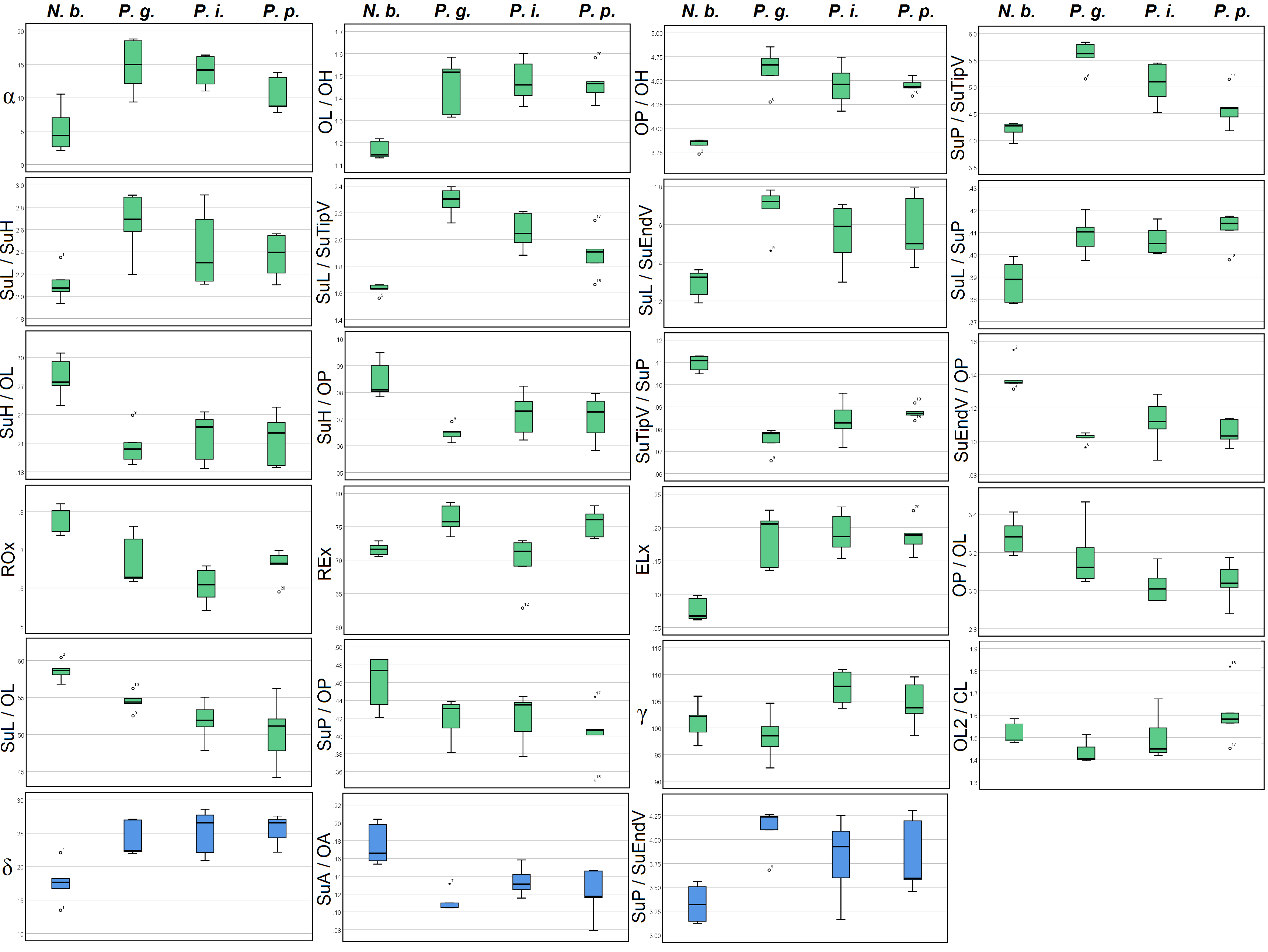

Supplement: S5 Fig — P. g., Ponticola gorlap; P. i., Ponticola iranicus; P. p., Ponticola patimari. Green, normally distributed variables; blue, non-normally distributed variables. (TIF) [file pone.0285857.s006.tif]

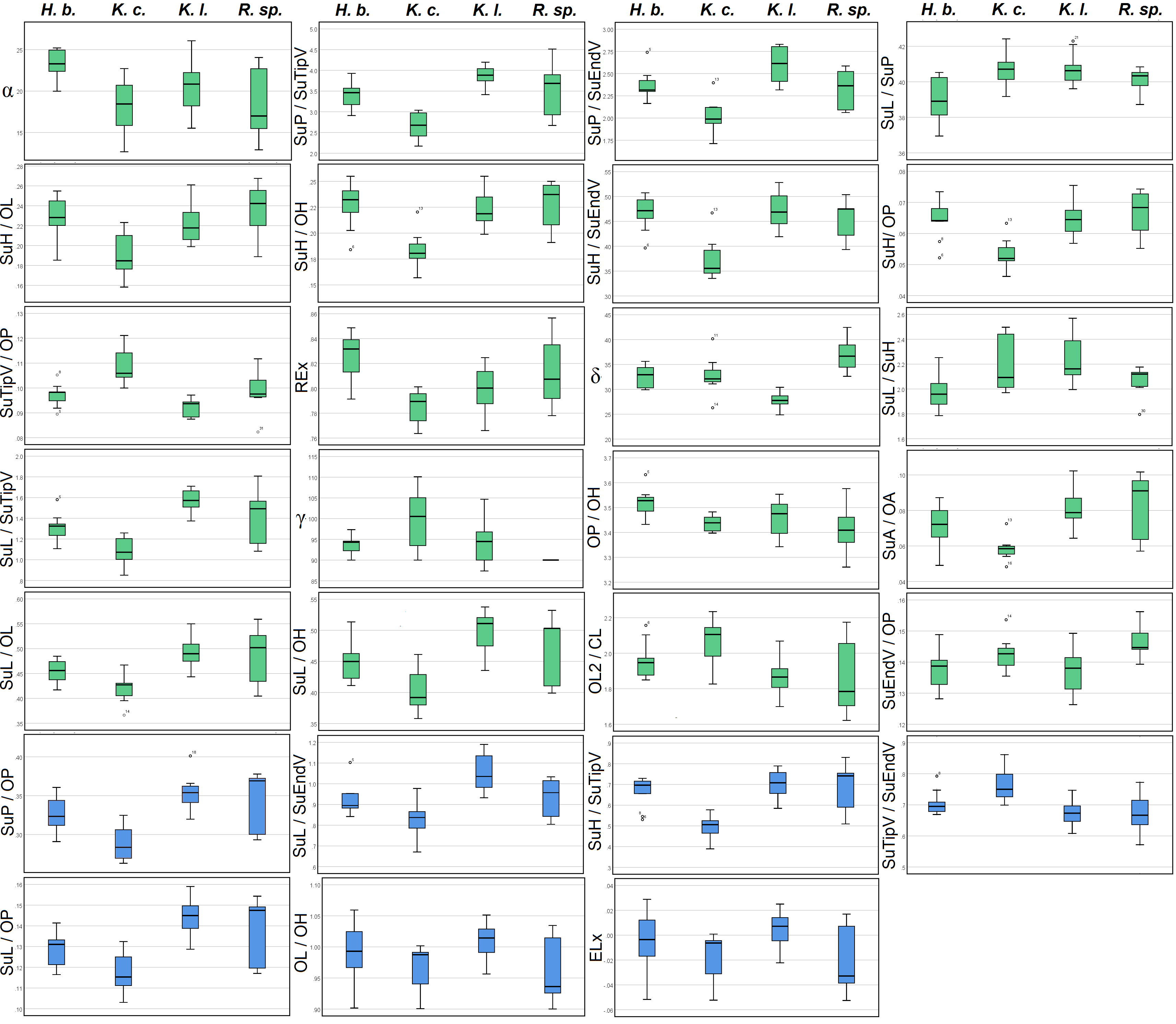

Supplement: S6 Fig — H. b., Hyrcanogobius bergi; K. c., Knipowitschia caucasica; K. l., Knipowitschia longecaudata; R. sp., Rhinogobius sp. Green, normally distributed variables; blue, non-normally distributed variables. (TIF) [file pone.0285857.s007.tif]
